# Dynamic changes in whole genome DNA methylation, chromatin and gene expression during mouse lens differentiation

**DOI:** 10.1186/s13072-023-00478-7

**Published:** 2023-01-25

**Authors:** William Chang, Yilin Zhao, Danielle Rayêe, Qing Xie, Masako Suzuki, Deyou Zheng, Ales Cvekl

**Affiliations:** 1 Department of Ophthalmology and Visual Sciences, Albert Einstein College of Medicine, Bronx, NY 10461 USA; 2 Genetics, Albert Einstein College of Medicine, Bronx, NY 10461 USA; 3 Present Address: University of California Santa Cruz, Santa Cruz, CA 95064 USA; 4 Neurology and Neuroscience, Albert Einstein College of Medicine, Bronx, NY 10461 USA

**Keywords:** ATAC-seq, Differentiation, DNA methylation, Gene regulation, Histone H3.3, Lens, Open chromatin, Pax6, RNA-seq

## Abstract

**Background:**

Cellular differentiation is marked by temporally and spatially coordinated gene expression regulated at multiple levels. DNA methylation represents a universal mechanism to control chromatin organization and its accessibility. Cytosine methylation of CpG dinucleotides regulates binding of methylation-sensitive DNA-binding transcription factors within regulatory regions of transcription, including promoters and distal enhancers. Ocular lens differentiation represents an advantageous model system to examine these processes as lens comprises only two cell types, the proliferating lens epithelium and postmitotic lens fiber cells all originating from the epithelium.

**Results:**

Using whole genome bisulfite sequencing (WGBS) and microdissected lenses, we investigated dynamics of DNA methylation and chromatin changes during mouse lens fiber and epithelium differentiation between embryos (E14.5) and newborns (P0.5). Histone H3.3 variant chromatin landscapes were also generated for both P0.5 lens epithelium and fibers by chromatin immunoprecipitation followed by next generation sequencing (ChIP-seq). Tissue-specific features of DNA methylation patterns are demonstrated via comparative studies with embryonic stem (ES) cells and neural progenitor cells (NPCs) at *Nanog*, *Pou5f1*, *Sox2*, *Pax6* and *Six3* loci. Comparisons with ATAC-seq and RNA-seq data demonstrate that reduced methylation is associated with increased expression of fiber cell abundant genes, including crystallins, intermediate filament (Bfsp1 and Bfsp2) and gap junction proteins (Gja3 and Gja8), marked by high levels of histone H3.3 within their transcribed regions. Interestingly, Pax6-binding sites exhibited predominantly DNA hypomethylation in lens chromatin. In vitro binding of Pax6 proteins showed Pax6’s ability to interact with sites containing one or two methylated CpG dinucleotides.

**Conclusions:**

Our study has generated the first data on methylation changes between two different stages of mammalian lens development and linked these data with chromatin accessibility maps, presence of histone H3.3 and gene expression. Reduced DNA methylation correlates with expression of important genes involved in lens morphogenesis and lens fiber cell differentiation.

**Supplementary Information:**

The online version contains supplementary material available at 10.1186/s13072-023-00478-7.

## Introduction

Cellular differentiation is driven by an intricate system of spatiotemporal regulation of transcription. Lineage-specific DNA-binding transcription factors bind to promoters and distal enhancers of individual genes that drive tissue-specific gene expression of a plethora of target genes; with many of them required to establish novel cellular phenotype. These processes are tightly linked to chromatin organization and its dynamic changes to promote or repress transcription of individual genes by enabling transcription factors to recognize their target sites [1, 2]. The accessible and restricted chromatin domains are referred as “open” and “closed” chromatin, respectively [3]. Despite major progress in this field using unbiased multi-omics approaches [4–6], many open questions, particularly related to chromatin landscape dynamics between inner cell mass/ES cells, early common progenitors, lineage-committed precursor cells and in terminally differentiating cells still exist.

The epigenetic regulatory mechanisms of cell type identity and maintenance include DNA methylation at cytosine residues, particularly at the CpG dinucleotides [7, 8], stable binding of transcription factors and other proteins such as Brd4 during mitosis (“mitotic bookmarking”), and distribution of specific histone modifications and core and linker histone variants [9–12]. Increased DNA methylation is often associated with chromatin compaction and reduced accessibility of chromatin for transcription factors and their associated chromatin remodeling complexes [8, 13, 14]. Importantly, binding of several transcription factors, such as CTCF, CREB, Jun, Nrf1, and Sp1 is impaired by methylation of cytosines [15, 16]. Thus, low-methylated regions are generally found in both promoters and enhancers of transcriptionally active genes [17]. During early mouse embryogenesis, DNA demethylation catalyzed by Tet methylcytosine dioxygenases (Tet1/2/3) [18, 19] regulates formation of germ layers and early cell-specific lineage formations while DNA methylation catalyzed by DNA methyltransferases (Dnmt1, Dnmt2, Dnmt3a and Dnmt3b) [20] controls de novo methylation patterns associated with cellular differentiation. Identification of DNA methylation patterns between multiple states of cellular differentiation is pivotal for our understanding of the epigenomic landscape and its role to control gene expression.

Histone H3.3 variants of the H3 histone family, including H3.1, H3.2 and H3.3 [21], are encoded by two genes located outside of the histone cluster genes [22, 23]. Transcriptionally active genes incorporate H3.3 histone variants independently of DNA replication [24–28]. Thus, studies of H3.3 localization are important for our comprehensive understanding of active chromatin landscape dynamics [28].

Embryonic eye development generates a plethora of highly specialized neuronal (e.g., photoreceptors and retinal ganglion cells) and non-neuronal (e.g., lens, cornea and trabecular meshwork) cells. Epigenetic mechanisms of eye and retinal development were examined through loss-of-function experiments of enzymes that control DNA methylation and demethylation and ATP-dependent chromatin remodeling [29]. Major differences in cytosine methylation were found between mouse cone and rod photoreceptors [30]. Ocular lens differentiation represents a powerful model to probe transcriptional regulatory mechanisms as expression of crystallin genes ranks among the most highly expressed genes in mammalian cells [31]. Lens formation from the anterior surface ectoderm represents a dynamic process orchestrated by lineage-specific transcription factors such as Pax6, Six3, Sox2, FoxE3, Prox1, Sox1, Gata3, and Hsf4 and common signaling pathways such as BMP, FGF, Notch, and Wnt [32]. Early lens formation is detectable as thickening of surface ectoderm above the approaching optic vesicle followed by formation of the lens placode and reciprocal invaginations to generate lens vesicle and optic cup. The anterior portion of the lens vesicle generates lens epithelium while its posterior portion undergoes massive cell elongation to fill the vesicle lumen and to generate primary lens fibers [33]. Lens growth is further driven by cell cycle exit-coupled differentiation of the equatorial epithelial cells to produce secondary lens fibers. Thus, the mature lens comprised a lens epithelium compartment containing domains of proliferating cells while the bulk of the lens is represented by the terminally differentiated lens fiber cell mass [32, 34, 35].

Three hallmark features characterize lens fiber cell differentiation. First, fiber cells accumulate very high amounts of lens-specific crystallin proteins required for lens transparency and refraction [35]. Second, lens fiber cells express unique lens-specific intermediate filament proteins filensin (Bfsp1) and phakinin (Bfsp2) [36], abundant cell membrane gap junction proteins Gja1, Gja3 and Gja8 [37], and lens-specific transmembrane proteins Mip/aquaporin 0 [38] and Lim2 [39]. Third, lens fiber cells degrade their intracellular organelles including nuclei to prevent light scattering [40–42]. How these lens-specific regulatory genes and their lens-specific targets are turned on in lens remain unknown although a wealth of transcriptomics data exist and can now be correlated with landscapes of chromatin accessibility both in mouse [43, 44] and chick [45, 46]. Recently, microdissected day 13 embryonic chick lenses into epithelium and fiber cell compartments were analyzed at three levels, including DNA methylation, open chromatin and transcription [47].

While earlier DNA methylation studies in lens were focused on promoter methylation in chicken and rat crystallin gene promoters [48–52], more recent studies have shown that the human αA-crystallin locus (CRYAA) is differentially methylated between control and age-onset cataract samples [53, 54]. Importantly, age-dependent DNA methylation reduces expression of genes involved in the antioxidant responses in mammalian lenses [55, 56]. Recently, loss-of-function studies of Dnmt1, Dnmt3a and Dnmt3b were conducted at different early stages of mouse lens morphogenesis [57]. Surprisingly, Dnmt1 inactivation at the lens placode stage cause apoptosis within the presumptive lens epithelium and generated only subtle abnormalities of the lens fibers. Conditional inactivation of both Dnmt3a and Dnmt3b in later stages of lens formation did not disrupt fundamentals of lens embryonic morphogenesis [57]. Thus, DNA methylation patterns established prior to the conditional inactivation of these three enzymes are sufficient to maintain lens cell type and control expression of individual genes within the differentiating lens.

To directly test this model, whole genome DNA methylation studies of the mouse lens differentiation are thus needed to identify differentially methylated regions (DMRs) and their association with chromatin accessibility and transcription. Herein, we performed WGBS using microdissected lenses from E14.5 embryos and newborn (P0.5) lenses. For comparative analyses, we used both DNA methylation data for embryonic stem (ES) (GEO accession GSE82125) and neuronal progenitor cells (NPCs) generated earlier [58] and our earlier RNA-seq and ATAC-seq data for the same lens microdissected samples [43, 44]. Our analyses also included mapping of histone H3.3 landscape and DNA methylation levels in Pax6-bound chromatin as well as in vitro analyses of Pax6 binding to their binding sites containing one or two methylated CpG dinucleotides. Taken together, these integrated data on lens chromatin landscape provide new insights into the expression of many genes encoding critical proteins for lens development and differentiation.

## Results

### Global analysis of DNA methylation during lens differentiation: reproducibility and distinctiveness of methylation in lens cells

**Fig. 1:**
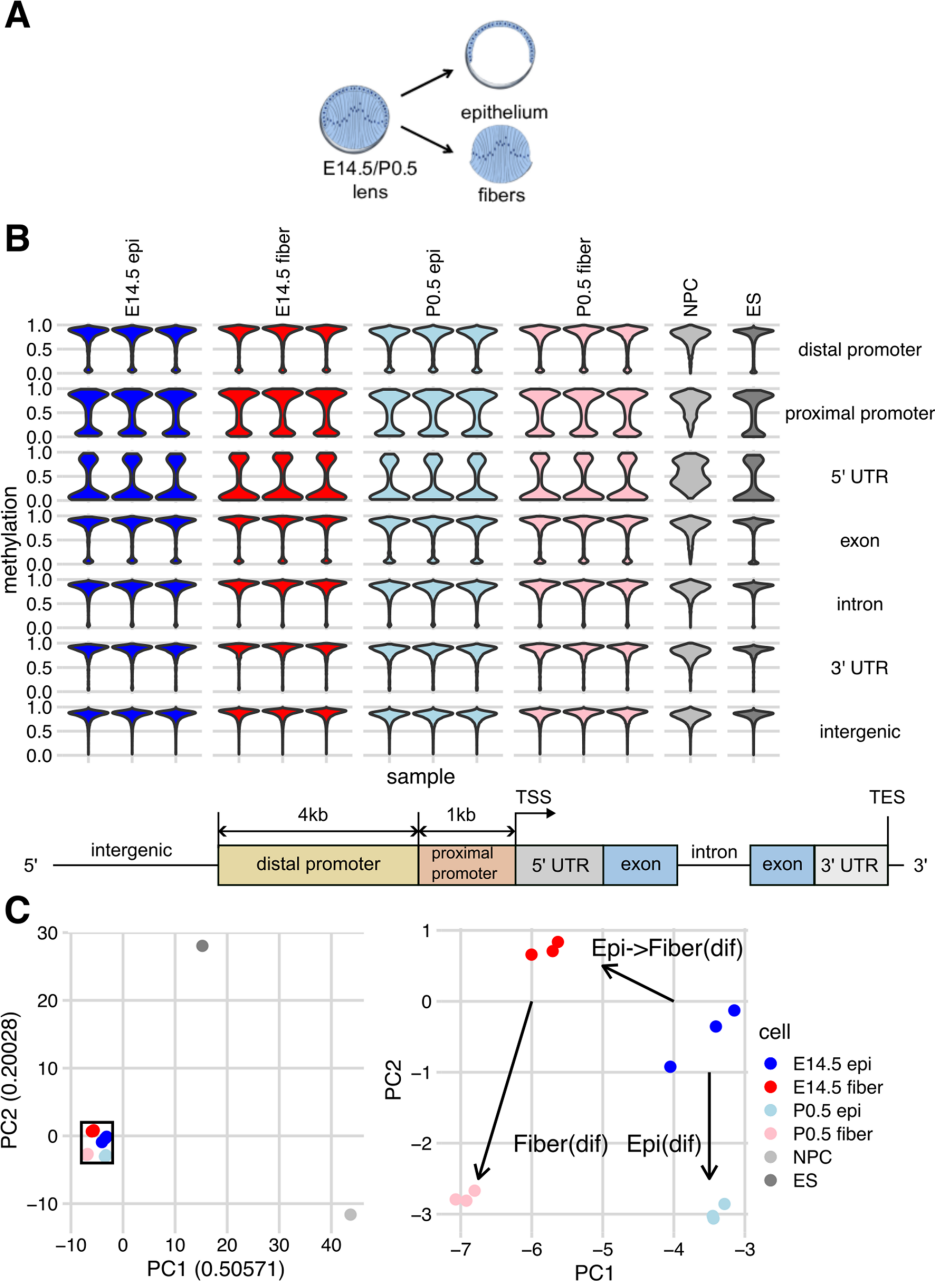
Experimental model and clustering of samples based on DNA methylation patterns using 100,000 randomly sampled CpGs. **A** Schematic illustration of lens epithelial and fiber cells compartments. **B** Violin plots showing distribution of CpG methylation scores across genomic features for all four groups lens samples and representative non-lens cells, including NPC and ES cells. Bottom panel: schematic of genomic feature annotations. **C** Left: PCA showing distinction in methylation patterns of sampled CpGs among lens samples and representative non-lens cells. Right: magnification of inset from left panel showing the three developmental path segments, path “epithelial” differentiation, Epi(dif), path “epithelial to fiber cell” differentiation at E14.5, EpiFiber(dif), and path “fiber cell” differentiation, Fiber(dif), respectively. The color codes of each sample are shown on the right

We collected lens epithelium and lens fibers from embryonic E14.5 and newborn (P0.5) mouse (CD1 strain, Charles River Laboratories) lenses (Fig. 1A), as described in our previous studies [43, 44], and performed WGBS using three biological replicates (see Methods). The data were compared to genome annotation, including intergenic, distal promoter, proximal promoter, 5′ untranslated region (5′UTR), exon, intron and 3′ UTR DNA sequences (Fig. 1B, see Materials and methods). Surveying 100,000 randomly sampled CpGs, we found that global CpG-level methylation was broadly similar across lens and ES cells, with a minority of CpGs hypomethylated across distal promoters, proximal promoters, and exons, with a majority of CpGs having low- or un-methylated regions across 5′UTRs. In contrast, almost fully methylated DNA was found in introns, 3′ UTRs, and intergenic regions. The NPC dataset, by comparison, showed less obvious unmethylation in promoters and 5′ UTRs (Fig. 1B). In summary, our results displayed as violin plots show that in lens and non-lens cells hypomethylation occurs predominantly in promoter regions of genes, followed by individual exons.

We next performed principal component analysis (PCA) of methylation at the 100,000 randomly sampled CpGs to ascertain the reproducibility of methylation patterns between individual samples and visualize the separation of different cell types. As expected, we found that methylation patterns of the four lens cell samples are more similar to each other than to non-lens cell types, i.e., ES and NPC cells. Additionally, variation between samples of the same lens cell subtype is consistently smaller than the variation between different lens cell subtypes, showing cell identity is distinct on the methylation level (Fig. 1C). Based on established lens differentiation processes and our previous studies of lens chromatin [44], we defined three lens differentiation pathways: lens epithelium differentiation from E14.5 to P0.5 (Epi(dif), formerly path1 in [44]), epithelial to fiber cell differentiation at E14.5 (EpiFiber(dif), formerly path2a), and fiber cell” differentiation from E14.5 to P0.5 (Fiber(dif), formerly path2b).

### Analysis of DNA methylation at *Nanog*, *Pou5f1, Sox2* and *Pax6* loci in ES, NPC and lens cells

**Fig. 2:**
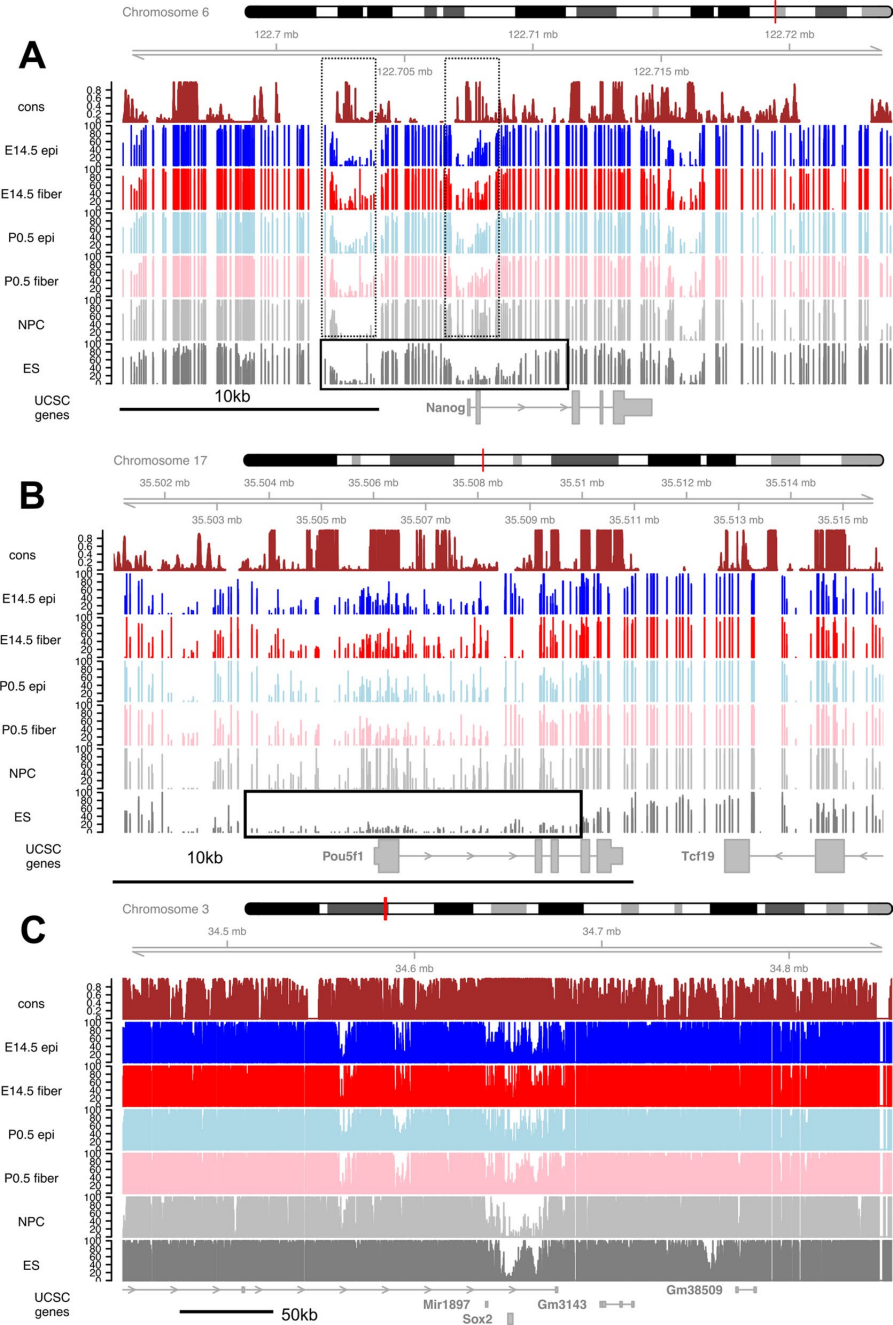
Comparison of methylation between lens and non-lens cells at the *Nanog*, *Pou5f1*, and *Sox2* loci encoding the core pluripotency GRN in the ES cells. **A** *Nanog* locus. We marked ~ 8 kb region of demethylation, including both the distal and proximal promoter regions and extending into the 5′-portion of intron 2 in ES cells (boxed). Gain of methylation in other cell types is also shown (dotted boxes). **B** *Pou5f1* locus. We marked ~ 6.5 kb of demethylated/low-methylated DNA that includes 5′-promoter flanking region extending towards intron 4 in ES cells (boxed). Major gain of DNA methylation is found in other samples (boxed). **C** *Sox2* locus. We show ~ 60 kb of Sox2 locus as this region contains multiple distal enhancers active during chicken lens placode formation [60]. Note that low methylation region (~ 8 kb) include upstream region of its promoter and the entire coding region of the *Sox2* locus

To demonstrate local changes in DNA methylation between core pluripotency genes in ES, NPC and lens cells, we show data for the Nanog, Pou5f1 (Oct3/4) and Sox2 loci, all three of them encoding sequence-specific DNA-binding transcription factors critical for the formation of the core pluripotency gene regulatory network (GRN) [59] (Fig. 2). In ES cell chromatin, the *Nanog* locus contains ~ 8 kb of a mixture of low and unmethylated regions flanked by regions of high methylation. Both the promoter and 5′-portion of intron 2 in ES cells show low CpG methylation while this region shows a major gain of methylation in NPCs and lens cells (Fig. 2A). The ~ 13-kb unmethylated/low-methylated domain of the *Pou5f1* locus spans across the 5′-promoter flanking region and intron 3 in ES cells and methylation is increased in both NPCs and lens tissues (Fig. 2B). Finally, the intron-less Sox2 gene is located within ~ 8 kb region with markedly reduced DNA methylation compared to its flanking genomic regions (Fig. 2C) that contain a large number of cell-specific distal enhancers, some active in the lens [60–63].

**Fig. 3:**
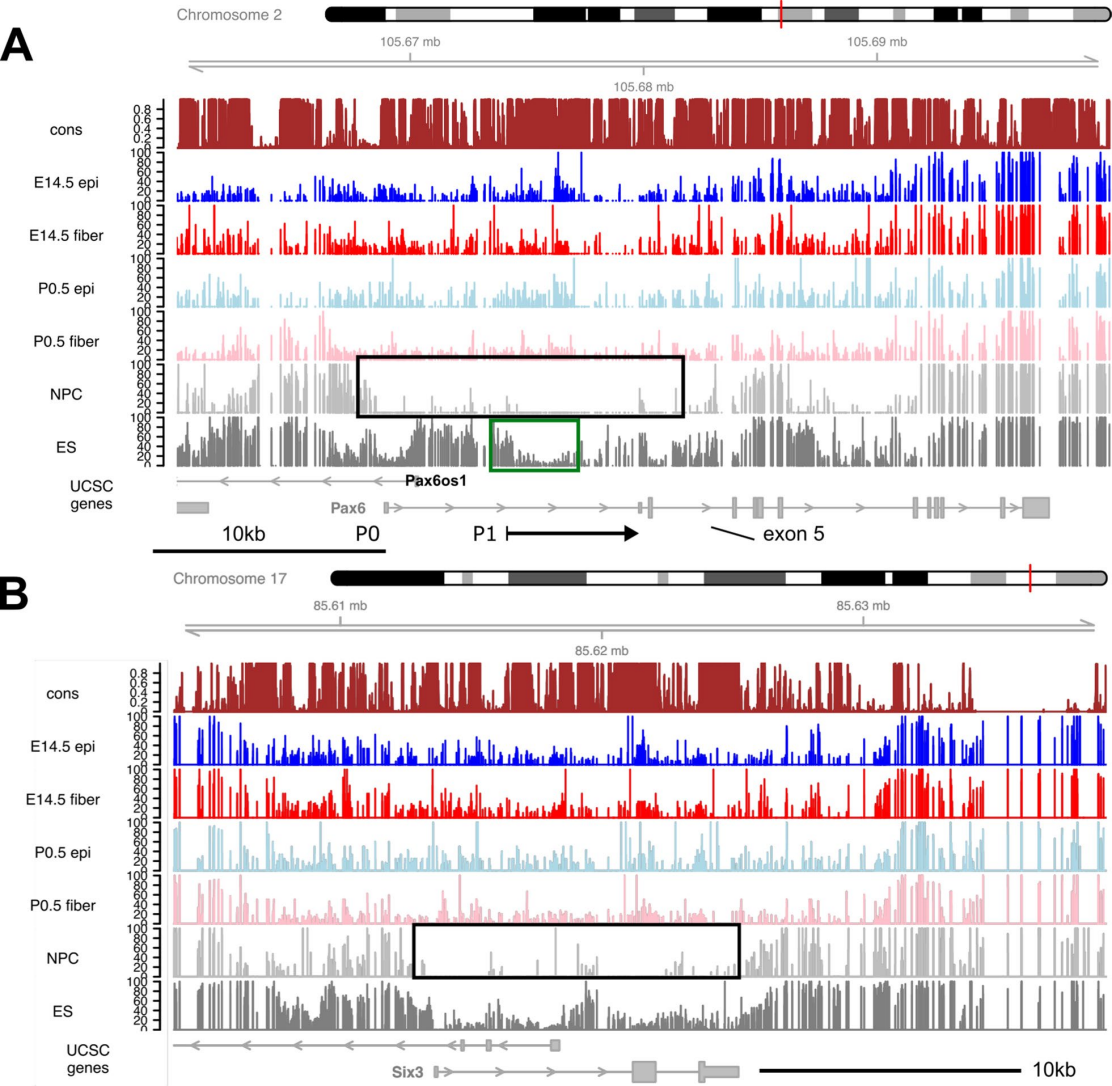
Comparison of methylation between lens and non-lens cells at *Pax6* and *Six3* loci. **A** *Pax6* locus including *Pax6os1*. All lens and NPC samples exhibit a broad continuous ~ 38 kb domain of reduced DNA methylation (boxed in the NPC track) in all four lens samples with the lowest signal across this region in E14.5 lens epithelium. **B** *Six3* locus including *Six3os1*. The region of low methylation in NPC is boxed. The individual tracks include evolutionarily conserved regions and DNA methylation in E14.5 lens epithelium (epi), E14.5 lens fibers, P0.5 lens epithelium (epi), P0.5 lens fibers, NPC and ES cells

In contrast, most genes encoding lineage-specific DNA-binding transcription factors driving the formation of new cell types are not expressed in ES cells [59]. The earliest stages of lens progenitor cells formation are regulated by Pax6, Six3 and Sox2 [63–68]. In lens, Pax6 functions as the earliest known and essential transcription factor-encoding gene for lens progenitor cell formation and all subsequent stages of lens differentiation, including GRN controlling crystallin gene expression [69]. Pax6 is also expressed in the early neuroectoderm [70, 71] and pancreas [72]. The mouse *Pax6* locus (total number of 15 exons) spans over 450 kb [73] and Fig. 3A shows only ~ 40 kb, including two Pax6 promoters: lens and retinal/brain promoters, P0 and P1, respectively [67]. In addition, an opposite promoter is found further upstream encoding lncRNA Pax6os1/Paupar [74]. As expected, the NPCs show much lower levels of DNA methylation including promoters P0 and P1 and downstream coding and noncoding regions compared to ES cells (Fig. 3A). All four lens samples show mixtures of low and moderately methylated domains clearly different from the NPCs suggesting general tissue-specific regulation of methylation at the *Pax6* locus (Fig. 3A). Note that our RNA-seq data found the highest levels of Pax6 expression in E14.5 lens epithelium compared to other samples analyzed here [43]. Detailed analysis of DMRs in lens chromatin at the *Pax6* locus is given below (section “DNA methylation and Pax6 binding).

The role of Six3 in lens cell formation compared to Pax6 is less well understood [65, 66] due to the multifunctionality of this transcription factor in earlier anterior neural plate and parallel optic vesicles formation [75]. Six3 is required for Pax6 expression in the head surface ectoderm [65], followed by reciprocal regulation of Six3 expression by Pax6 in the prospective lens ectoderm [65, 66]. Conditional inactivation of Six3 in the prospective lens ectoderm disrupts lens placode formation [66]. The present analysis shows reduced DNA methylation of the transcribed portion of the *Six3* locus in NPCs compared to ES cells (Fig. 3B), as well as gain of methylation across this region in all lens samples. Regarding dual roles of Sox2 in ES and lens placode GRNs, expression of Sox2 drops in the lens cells following the beginning of primary lens fiber cell formation concomitant with upregulation of Sox1 [76]. Correspondingly, gain of DNA methylation across the Sox2 coding region in E14.5 lens chromatin is observed (Fig. 2C). Taken together, these data identify major differences in DNA methylation between ES cells and NPCs and microdissected lens tissues at three representative critical loci that control pluripotency (Nanog, Pou5f1 and Sox2) and lens placode formation (Pax6, Six3 and Sox2).

### Identification of low and unmethylated regions during lens cell differentiation

**Fig. 4:**
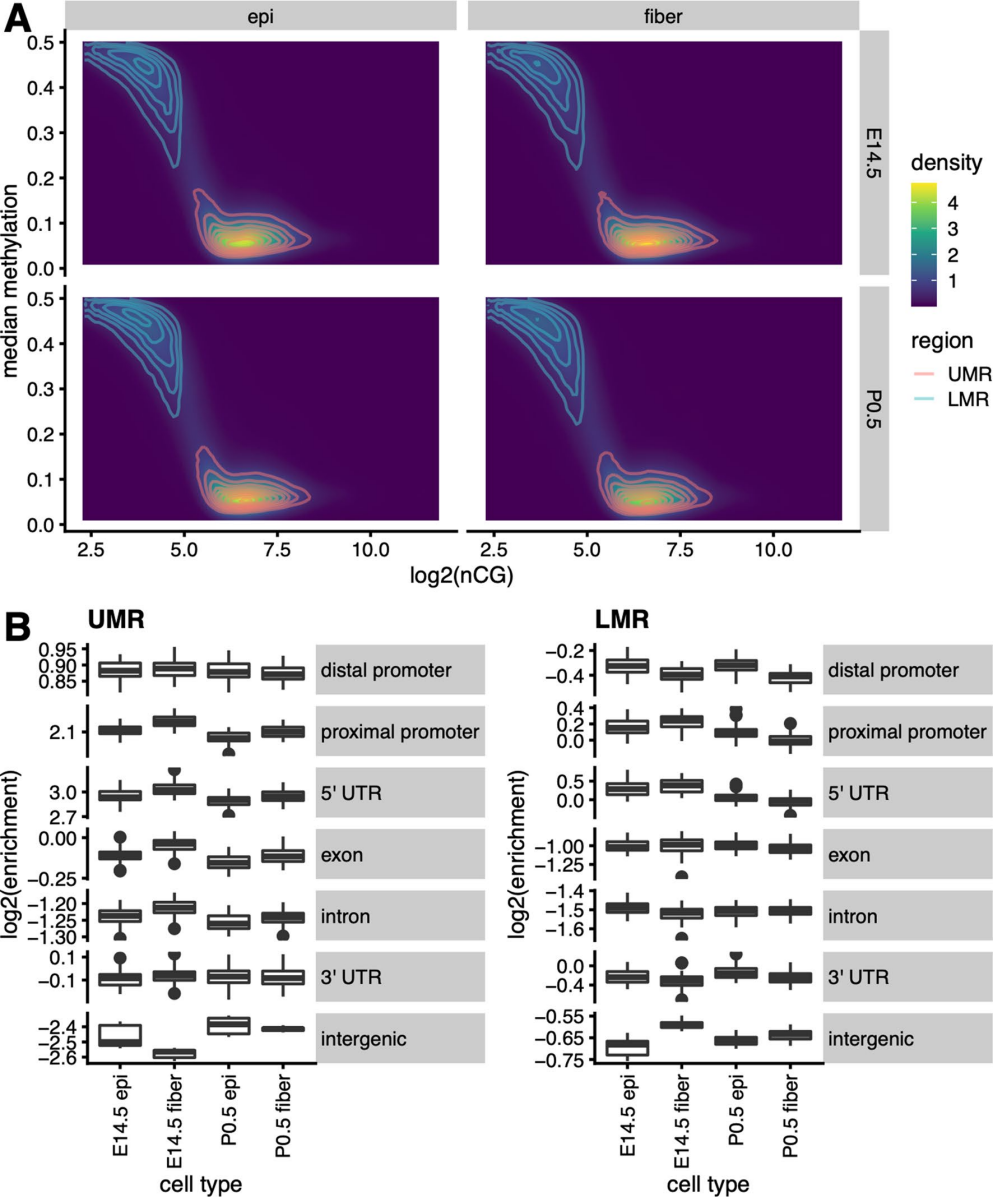
Identification and quantitative analyses of UMRs and LMRs in four lens samples. **A** Distributions of median methylation and number of CpGs in un- or low-methylated regions. Solid horizontal line within boxes: median of median per-region methylation scores. Bottom and top box edges: first and third quartiles, respectively. Bottom and top whiskers: data no smaller than 1.5× the interquartile range from the bottom edge and no greater than 1.5 × the interquartile range from the top edge, respectively. Points: outliers exceeding whisker values. **B** Log2 enrichment of both UMR and LMR regions across genomic features compared to 20 random shuffles of regions

Low methylation at promoters and regulatory regions is associated with increased gene expression [17]. We thus identified and characterized regions in the lens genome that were low in methylation compared to other genomic regions. As in previous studies [4–6], we defined two types of such regions, unmethylated regions (UMRs) and low-methylated regions (LMRs) (see Materials and methods), corresponding to regions of > 32 CpGs with median methylation < 20%, and those of < 32 CpGs with median methylation between 20 and 50%, respectively (Fig. 4A). We then calculated the enrichment of UMRs/LMRs across genomic features, defined as the fold change in the frequency of finding such regions located within a feature compared to what was obtained for 20 random shufflings of regions of the same number and size across the whole genome (Fig. 4B). We found UMRs to be enriched by 2- to 8-fold in distal and proximal promoter regions and 5′ UTRs, while depleted by < twofold in exons and 2- to 4-fold in introns and intergenic regions. LMRs showed a weaker enrichment and depletion, being enriched by < twofold in proximal promoters and 5′ UTRs, depleted by <twofold in distal promoters, 3′ UTRs, and intergenic regions, and depleted by approximately twofold in exons and introns (Fig. 4B). This is probably related to low numbers of CGs in those regions necessary for accurate quantification of DNA methylation. For the subsequent analyses, we focused only on regions that were reproducible across all three replicates of each lens cell type.

**Fig. 5:**
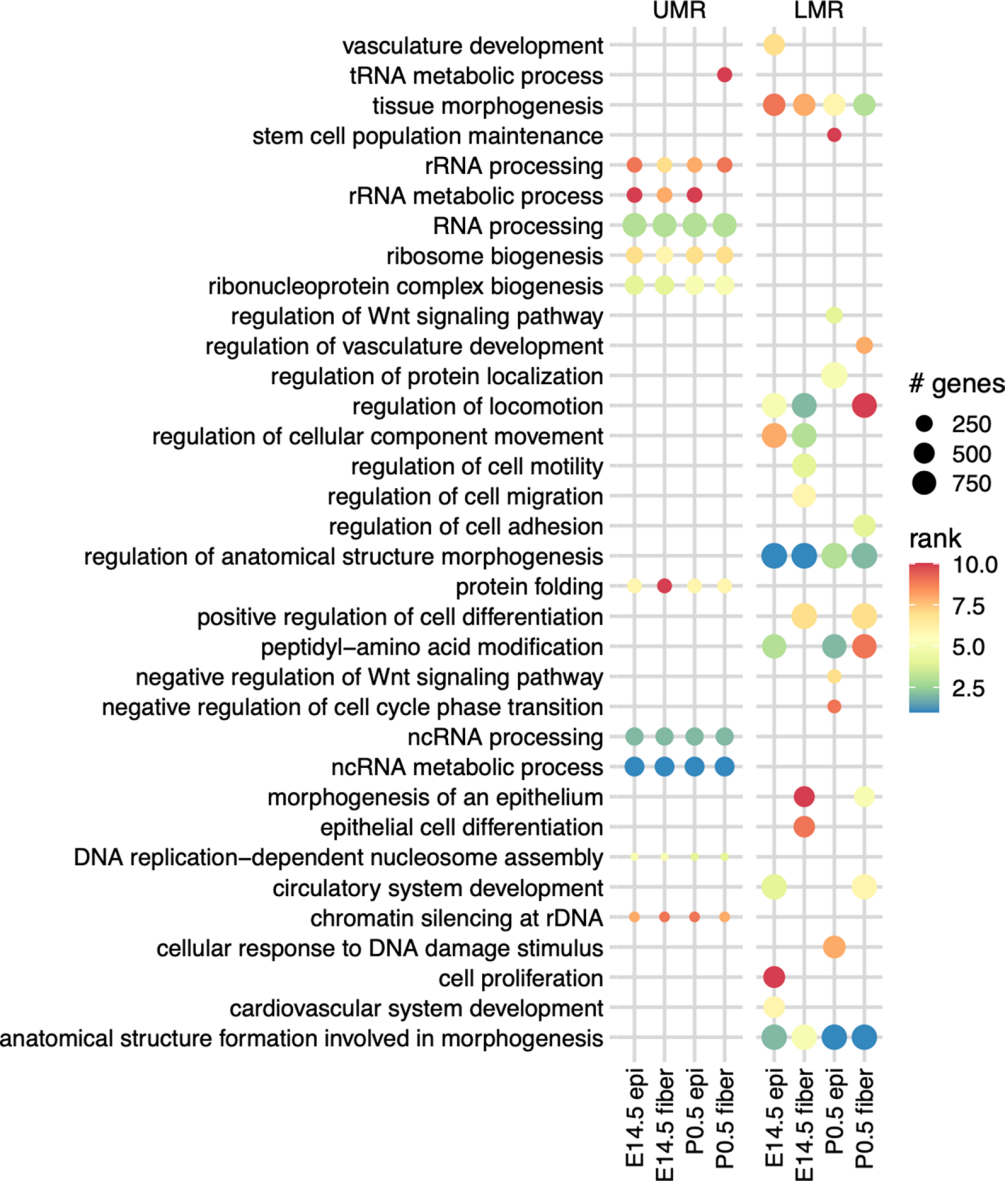
GO enrichment of reproducible UMRs and LMRs across four lens cell types. The figure shows the top 10 biological process terms in each cell type

To understand the functional impact of these low-methylated regions, we performed Gene Ontology (GO) enrichment analysis for each set of UMRs and LMRs. Comparing the top 10 most significant (by binomial p-value) GO biological process terms from each cell type, we found broad overlaps in common cell functions such as regulation of anatomical structure morphogenesis (for LMR) and RNA processing (for UMR), consistent with the view that genes important for universal cellular processes are unmethylated and transcriptionally active across cell types, including lens cells (Fig. 5). Individual genes from the top five groups are shown in Additional file 1. For the full result of GO analysis for LMRs, see Additional file 2.

### Differentially methylated regions during lens cell differentiation

**Fig. 6:**
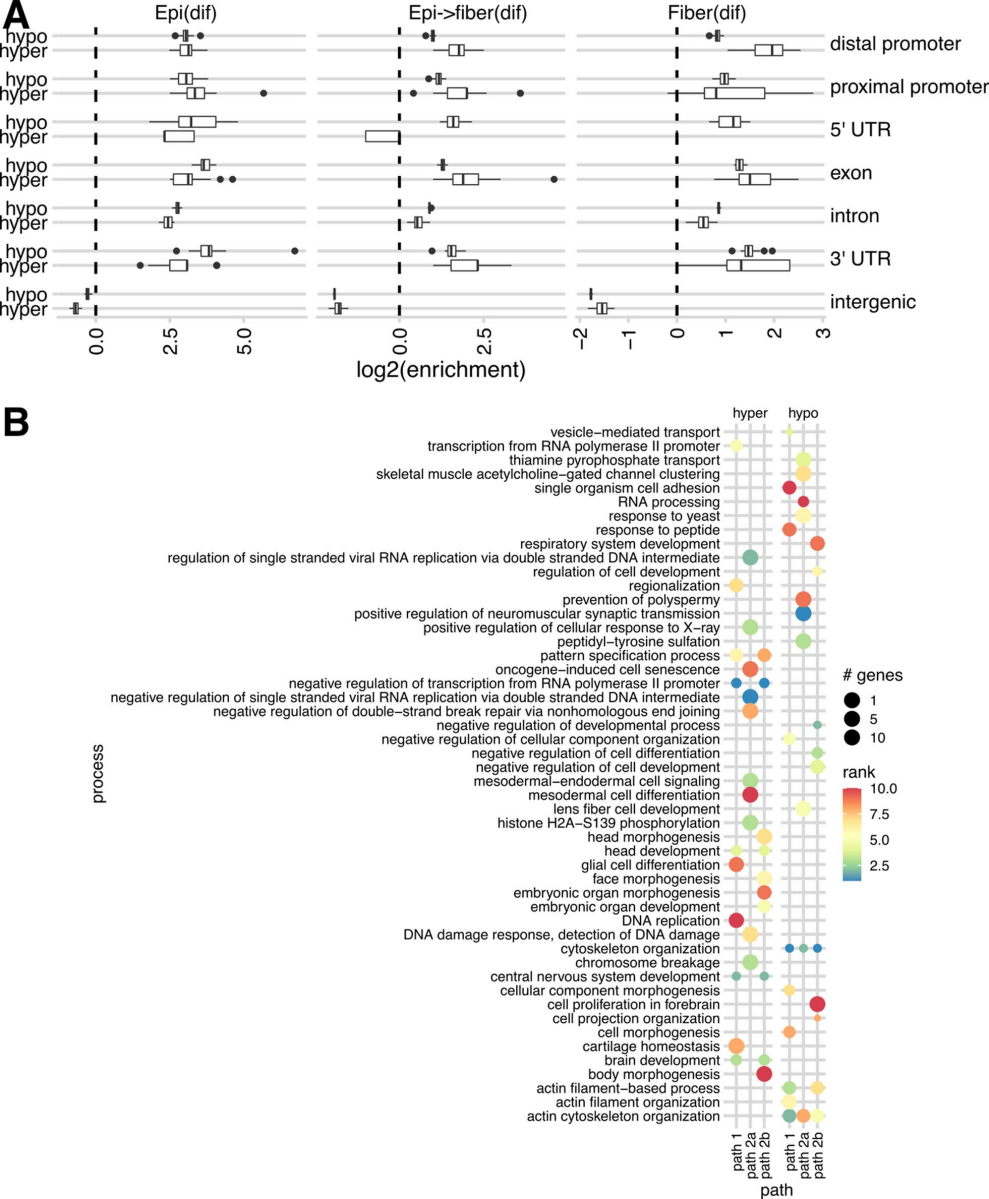
Regions of differential DNA methylation between three lens differentiation pathways: Epi(dif), EpiFiber(dif) and Fiber(dif). **A** Log2 enrichment of DMRs at different genomic features compared to 20 iterations of randomly shuffled regions for each differentiation path across genomic features. For details on the boxplot representation, see Fig. 3 caption. No enrichment (identical to random) is indicated with a dashed line. **B** GO enrichment of DMRs in each three individual paths

We next investigated whether these DNA-methylation changes play any role in the differentiation of lens cell types along each developmental path (see Fig. 1C), specifically in distinguishing lens fiber from epithelium, and newborn cells from embryonic cells of the same type. We thus determined DMRs between the “early” and “advanced” cell subtypes of each developmental path segment defined above: Epi(dif), EpiFiber(dif) and Fiber(dif). Similarly, to the above UMR/LMR analysis, we calculated enrichments of DMRs across genomic features against 20 iterations of randomized chosen regions. We found both hypermethylation and hypomethylation to be enriched in gene bodies and distal promoters and depleted in intergenic regions in all three path segments, indicating that methylation-driven regulatory mechanisms in lens cell differentiation are concentrated to gene bodies and both distal and proximal promoters (Fig. 6A).

To gain insights into functional consequences of DMRs in lens development, we next performed GO analysis on hypermethylated and hypomethylated DMRs for each path segment. We combined the top 10 enriched GO biological process terms for either hypermethylated or hypomethylated DMRs over each path for comparison. The biological process term “lens fiber cell development” appeared in EpiFiber(dif) path (Fig. 6B). This path represents most dramatic differentiation step, formation of lens fibers from lens epithelial cells. As with low-methylated regions, we found many biological process terms pertaining to shared cellular processes such as actin organization, morphogenesis, and DNA repair, all processes involved in elongation of lens fibers and maintenance of nuclei prior their denucleation [33]. Interestingly, we also found non-lens specific developmental terms, including those related to forebrain and glia, suggesting that differential methylation in lens regulates developmental processes shared among cells of ectodermal lineage (Fig. 6B). For the full result of the GO analysis of DMRs, see Additional file 3.

### Differential methylation and general mechanisms of epigenetic modifications

We further sought to understand how differential methylation integrated with other mechanisms of gene control at the level of chromatin. Herein, we generated ChIP-seq data of histone H3.3 variants (H3.3) in newborn microdissected lenses. In addition, we compared the present DNA methylation data with our previously published ChIP-seq data of global H3 histone K27 acetylation (H3K27ac) and histone H3 K4 monomethylation (H3K4me1) in whole newborn (P0.5) lenses [31].

#### ChIP-seq of histone H3.3 variant

**Fig. 7:**
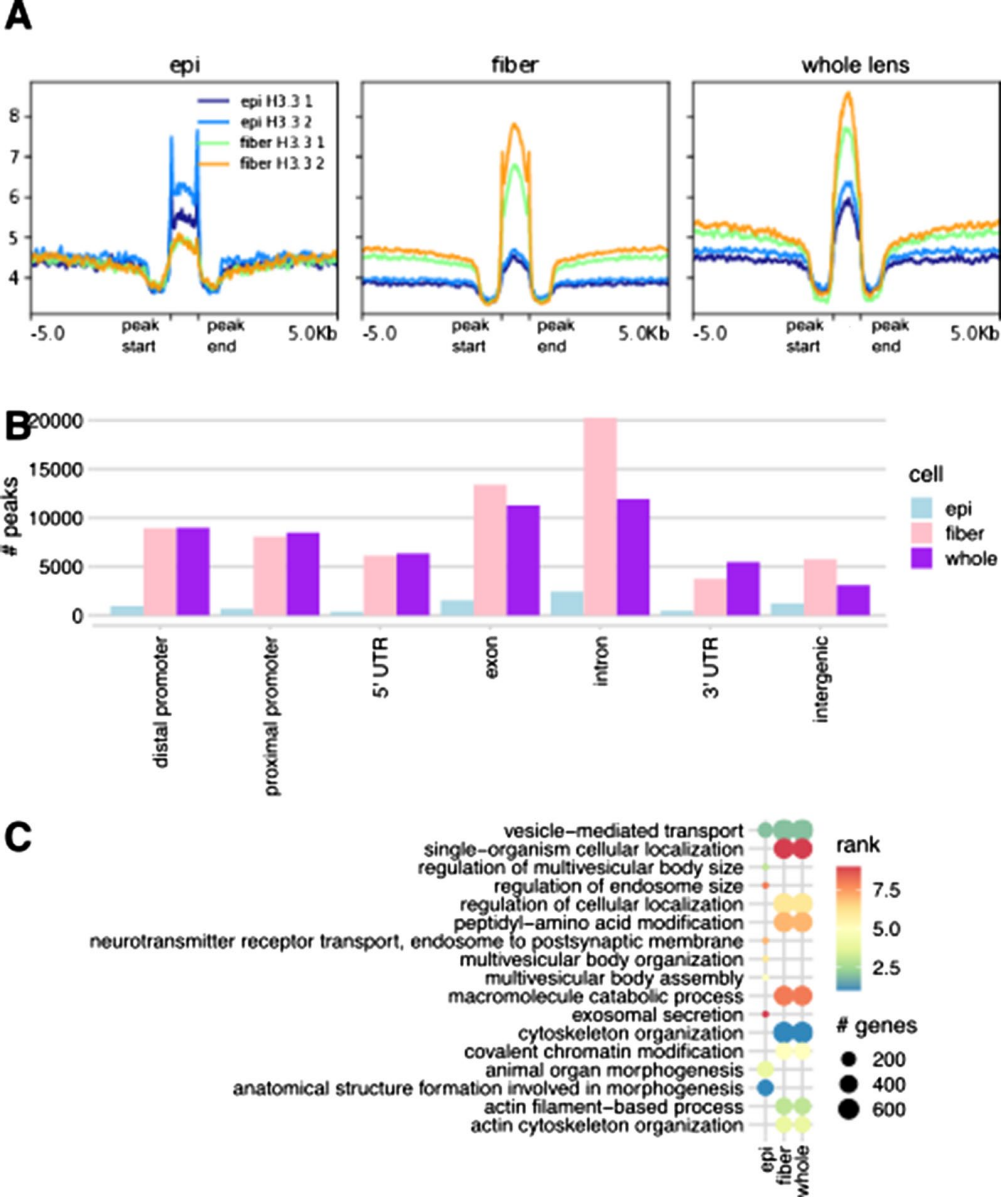
Global analysis of histone H3.3 variant in lens cell chromatin. **A** Aggregated histone H3.3 ChIP-seq read densities within ± 5 kb of called peaks for all four samples (two replicates each). **B** Numbers of histone H3.3 ChIP-seq peaks in newborn lens by genomic feature (see Fig. 1B), categorized as lens epithelium-specific, fiber-specific, or shared. **C** Top enriched GO biological process terms for three categories of histone H3.3 peaks

In this initial phase of integrative epigenetic analysis, we analyze the new histone H3.3 ChIP-seq data. Overall, we found the greatest number of H3.3 peaks in newborn lens fiber cells, followed by peaks shared between epithelium and fiber, and lower number of epithelium-specific peaks. In both epithelium and fibers, the H3.3 ChIP-seq signals were higher within cell-specific peaks compared to surrounding areas; however, peak intensity was overall higher in lens fibers (Fig. 7A). In shared peaks, mean signal was higher in fiber cells compared to epithelium (1.26-fold increase). This suggests that newborn epithelium and fibers largely share the same H3.3 peaks with the signal being more prominent or robust in fiber (Fig. 7A) consistent with higher levels of transcription of genes encoding lens structural and architectural proteins in fiber cells. As histone H3.3 is generally enriched at actively transcribed regions [24–28], its peaks were less frequently found in 3′ UTRs and intergenic regions compared to other genomic features (Fig. 7B, Table 1).

**Table 1:** Histone H3.3 ChIP-seq peaks across genomic features

Genomic feature	Epithelium-specific H3.3 peaks	Fiber-specific H3.3 peaks	Common H3.3 peaks
Distal promoter	954	8941	8988
Proximal promoter	674	8077	8487
5′ UTR	375	6126	6358
Exon	1545	13,390	11,282
Intron	2442	20,249	11,928
3′ UTR	473	3749	5470
Intergenic	1234	5746	3103

Counts of peaks in histone H3.3 density across annotated genomic features. Fewer epithelium-specific peaks were observed compared to fiber-specific or shared peaks

We next performed three independent GO analyses of epithelium-specific, fiber-specific, and shared histone H3.3 peaks. We found the top biological process terms to be identical for fiber-specific and shared peaks, further supporting the hypothesis that H3.3 regulates the same set of biological processes in epithelium and fiber. Several epithelium-specific biological process terms also emerged, including two terms related to organ morphogenesis; however, the epithelium-specific analysis is less statistically significant due to the small number of epithelium-specific peaks (Fig. 7C). For the full result of the GO analysis of H3.3 peaks, see Additional file 4. Individual genes from “Regulation of cellular organization”, “Macromolecule catabolic process”, “Cytoskeleton organization”, “Actin filament-based process” and “Actin cytoskeleton organization” groups from paths Epi(dif), EpiFiber(dif) and Fiber(dif) are shown in Additional file 5.

#### Cross-analysis of differential DNA methylation and histone H3K27ac and H3K4me1 modifications

**Fig. 8:**
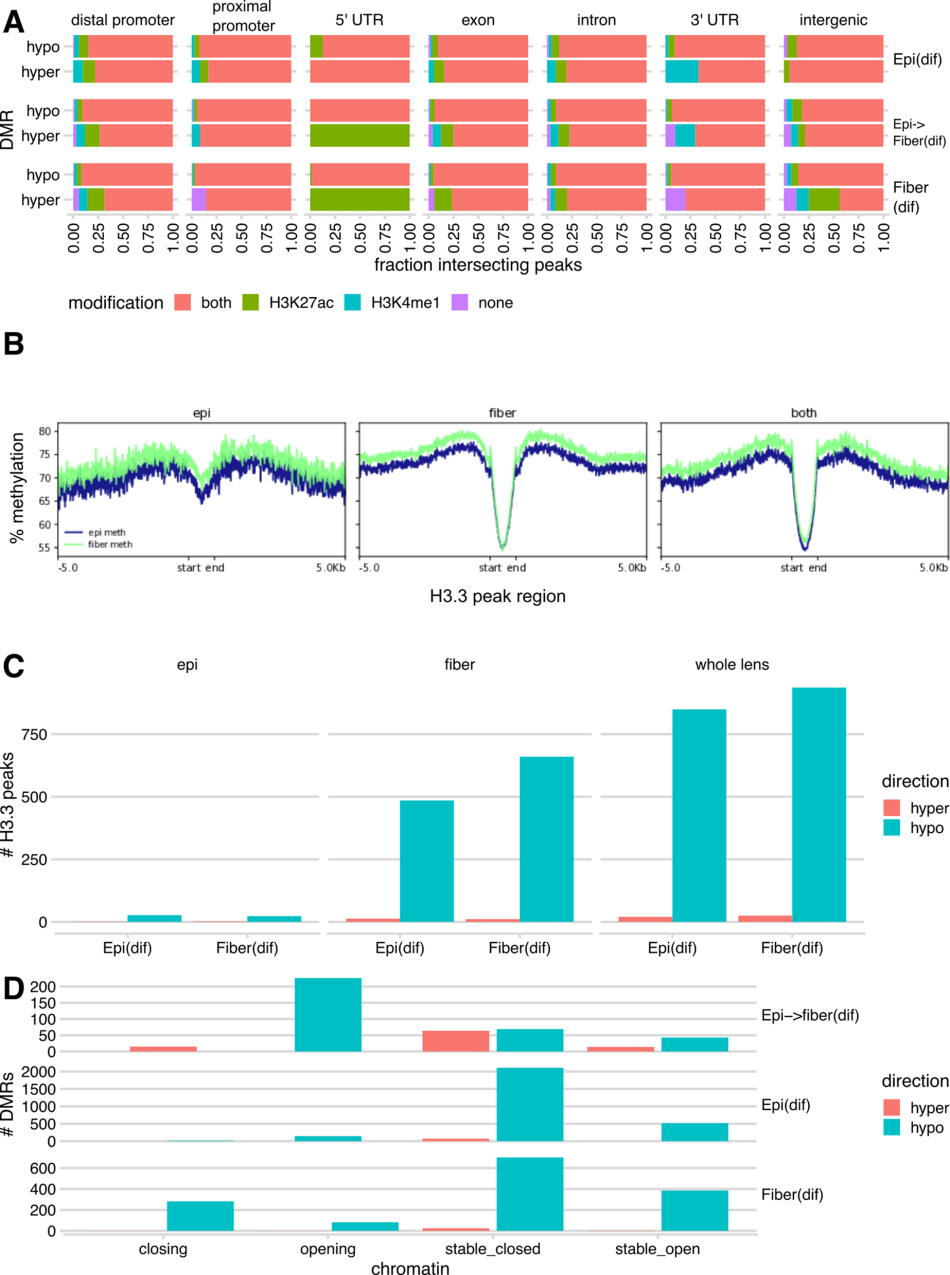
Relationships between DNA methylation, histone H3K27ac and H3K4me1 modifications, and H3.3. **A** Fractions of DMRs intersecting with H3K27ac or H3K4me1 ChIP-seq peaks from whole newborn lens chromatin [31]. **B** % methylation within ± 5 kb of newborn epithelium-specific, fiber-specific, and shared H3.3 peaks. **C** Counts of DMRs intersecting H3.3 peaks across the Epi(dif) and Fiber(dif) paths. **D** Counts of DMRs intersecting chromatin states across all paths

It has been shown that a combination of H3K4me1 and H3K27ac modifications predicts active enhancers while these individual modifications predict poised enhancers [77, 78]. We next examined the association between DMRs, histone modifications, and histone variants through identification of intersections between DMRs, previously identified peaks of H3K27ac and H3K4me1 modifications from newborn whole mouse lens [31] and the present H3.3 ChIP-eq data. We found that, across all three developmental paths Epi(dif), EpiFiber(dif) and Fiber(dif), most of both hypo- and hypermethylated DMRs across all genomic features intersected with both H3K27ac and H3K4me1 peaks, showing that differential DNA methylation and histone modification target the same genomic regions in lens chromatin (Fig. 8A).

Examining the relationship between differential DNA methylation and H3.3 variants in newborn microdissected lens epithelium and lens fiber, we found that DNA methylation levels were lower within H3.3 peaks compared to surrounding regions in both epithelium and fibers, including both cell-specific and shared peaks (Fig. 8B). This further suggests that many genomic regions are regulated by both demethylation and transcriptionally dependent histone H3.3 incorporation in lens chromatin.

A minority of histone H3.3 peaks in newborn lens epithelium and fiber cells intersected with DMRs from developmental paths Epi(dif) and Fiber(dif), mostly within hypomethylated DMRs (Fig. 8C and Table 2). Together with the observation that methylation is decreased within H3.3 peaks compared to surrounding regions in both newborn lens epithelium and fiber, this suggests that a majority of regions corresponding to H3.3 peaks in newborn epithelium and fiber are already demethylated in embryonic epithelium and fiber, respectively. The remaining regions are demethylated during maturation from the embryonic to the newborn stage in both the epithelial and fiber compartments.

**Table 2:** Counts of histone H3.3 ChIP-seq peaks intersecting DMRs

DMRs	Epithelium-specific H3.3 peaks	Fiber-specific H3.3 peaks	Common H3.3 peaks
None	3678	23,462	8968
Epi(dif) hypermethylated	1	13	20
Epi(dif) Hypomethylated	27	485	849
Fiber(dif) hypermethylated	2	11	25
Fiber(dif) hypomethylated	23	660	936

Counts of intersections between H3.3 peaks and DMRs categorized by H3.3 peak cell specificity and DMR developmental path segment and direction of methylation change

#### Comparative analysis of differential DNA methylation and chromatin accessibility

Changes in DNA methylation are often associated with changes in chromatin accessibility [8, 13, 14]. We thus compared differential DNA methylation and changes in chromatin accessibility along each developmental path Epi(dif), EpiFiber(dif) and Fiber(dif) segment, using our previously published ATAC-seq data [44]. Intersecting DMRs with differentially accessible regions (DARs), we found hundreds of hypomethylated DMRs in all three paths intersecting with opening chromatin, with a much smaller number of hypermethylated DMRs intersecting with closing chromatin. Since most of the DMRs are hypomethylated, this is as expected. However, for path EpiFiber(dif) and Fiber(dif), we observed a subset of hypomethylated DMRs intersecting with closing chromatin, suggesting a role of additional mechanisms driving changes in chromatin conformation. Finally, most DMRs, particularly those from paths EpiFiber(dif) and Fiber(dif), did not intersect with DARs (Fig. 8D and Table 3).

**Table 3:** Differentially methylated and accessible regions across paths and genomic features

Path	DMR direction	Chromatin	Distal promoter	Proximal promoter	5′ UTR	Exon	intron	3′ UTR	Intergenic
Epi(dif)	Hyper	Closing	1	1	0	0	1	0	0
Epi(dif)	Hyper	Opening	0	0	0	0	0	0	0
Epi(dif)	Hyper	Stable_closed	32	11	1	24	76	8	11
Epi(dif)	Hyper	Stable_open	0	0	0	1	1	0	0
Epi(dif)	Hypo	Closing	5	2	0	5	13	2	0
Epi(dif)	Hypo	Opening	34	23	7	37	117	7	30
Epi(dif)	Hypo	Stable_closed	566	199	49	429	1814	132	255
Epi(dif)	Hypo	Stable_open	160	71	24	154	447	50	61
Epi- > fiber(dif)	Hyper	Closing	7	1	0	3	13	0	2
Epi- > fiber(dif)	Hyper	Opening	0	0	0	0	0	0	0
Epi- > fiber(dif)	Hyper	Stable_closed	21	7	2	10	51	1	6
Epi- > fiber(dif)	Hyper	Stable_open	4	1	0	2	14	0	0
Epi- > fiber(dif)	Hypo	Closing	0	0	0	0	0	0	0
Epi- > fiber(dif)	Hypo	Opening	82	28	4	81	219	21	16
Epi- > fiber(dif)	Hypo	Stable_closed	22	6	4	21	58	6	11
Epi- > fiber(dif)	Hypo	Stable_open	14	6	2	14	37	6	6
Fiber(dif)	Hyper	Closing	2	0	1	1	1	0	0
Fiber(dif)	Hyper	Opening	0	0	0	0	1	0	0
Fiber(dif)	Hyper	Stable_closed	10	1	0	4	14	1	7
Fiber(dif)	Hyper	Stable_open	1	0	0	2	2	1	0
Fiber(dif)	Hypo	Closing	95	36	7	83	254	23	20
Fiber(dif)	Hypo	Opening	21	10	5	36	73	12	10
Fiber(dif)	Hypo	Stable_closed	161	70	14	139	616	43	82
Fiber(dif)	Hypo	Stable_open	116	57	20	139	349	40	25

DMARs sorted by path segment, methylation change, chromatin accessibility change, counted across annotated genomic features

Across genomic features, DMRs that intersected with DARs distributed similarly to the full population of DMRs, with the majority residing in introns, but a significant minority found in 1–5 kb upstream, at promoters, and 5′ UTRs (Table 3). As described above, the sizes of regulatory regions upstream of the TSS will vary across genes as regions annotated as 1–5 kb upstream frequently serve regulatory functions as extended promoter regions and/or proximal enhancers.

#### Methylation, chromatin accessibility, and histone H3.3 distribution at representative crystallin loci

**Fig. 9:**
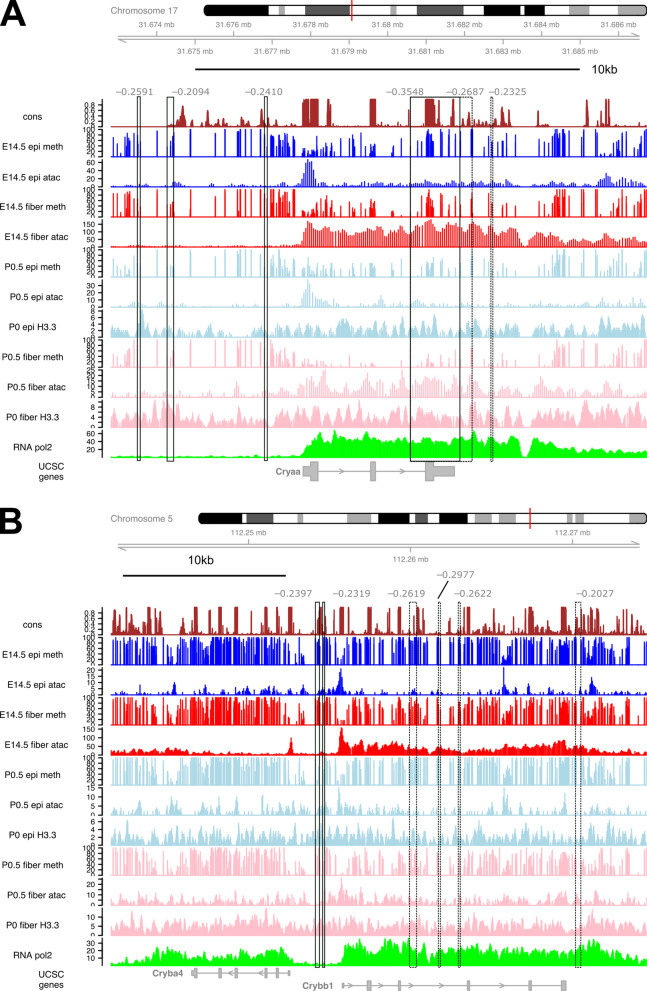
DNA methylation and chromatin accessibility profiles in the *Cryaa* and *Cryab4*-*Crybb1* loci. **A** *Cryaa* locus (chromosome 17). **B** *Cryba4*-*Crybb1* loci expressed in opposite directions (chromosome 5). In addition to four DNA methylation tracks (see Fig. 2), two H3.3 ChIP-seq and four ATAC-seq tracks are shown. The bottom track shows RNA polymerase II ChIP-seq data (whole lens) [79]. Boxes denote DMRs: solid lines: path Epi(dif); dashed lines: path EpiFiber(dif).

The αA-crystallin (Cryaa) is the most highly expressed gene in newborn lens fibers [43, 79] and shows abundant RNA polymerase II from the promoter, across the gene body and 3′-UTR in whole newborn lens chromatin [79]. Reduced DNA methylation all across the *Cryaa* locus, including far 3′-UTR is most notable in P0.5 lens fibers (Fig. 9A). Correspondingly, the *Cryaa* promoter region is open in E14.5 epithelium and the whole *Cryaa* locus is in open chromatin in the lens fibers (Fig. 9A). A weaker ATAC-seq signal further upstream corresponds to evolutionary conserved enhancer region [80]. As expected, histone H3.3 is more abundant at the Cryaa gene in P0.5 lens fibers compared to the epithelium (Fig. 9A). In contrast, the *Cryba4*-*Crybb1* bi-directional locus [81] is marked by methylation all across the coding regions with reduced methylation within the region harboring the promoters (Fig. 9B). Open chromatin domain of the *Crybb1* locus is larger compared to the *Cryba4* locus as the Crybb1 mRNAs are more abundant [43, 44]. Importantly, the histone H3.3 domains in general corresponds to the RNA Polymerase II distributions (Fig. 9B). Larger-scale genomic views of the *Cryaa* locus and a cluster of five γ-crystallin genes (*Cryga*, *Crygb*, *Crygc*, *Crygd* and *Cryge*), marked by abundant H3.3 signals in the lens fiber chromatin and RNA polymerase II (whole lens chromatin data), are shown in Additional file 6.

Gap junction proteins Gja1 (connexin43), Gja3 (connexin46) and Gja8 (connexin50) and Mip (major intrinsic protein/aquaporin 0) are major proteins expressed in the lens [82, 83]. Gja1 is already expressed in the lens placode and is enriched in lens epithelium. Correspondingly, the lowest DNA methylation in the promoter and downstream region is found in E14.5 epithelium compared to other three samples (Additional file 7). Gja8 is highly expressed in lens fibers, and, the lowest methylation is found in the promoter and downstream region in P0.5 lens fibers. Data for lens-specific intermediate filament proteins Bfsp1 and Bfsp2 are also shown. For comparisons, FoxE3 is a sequence-specific DNA-binding transcription factor expressed mostly in the lens epithelium [84, 85]. There are no major differences in DNA methylation between lens epithelium and lens fibers in the *Foxe3* locus (Additional file 7).

### Integration of epigenetic regulatory mechanisms with gene expression

**Fig. 10:**
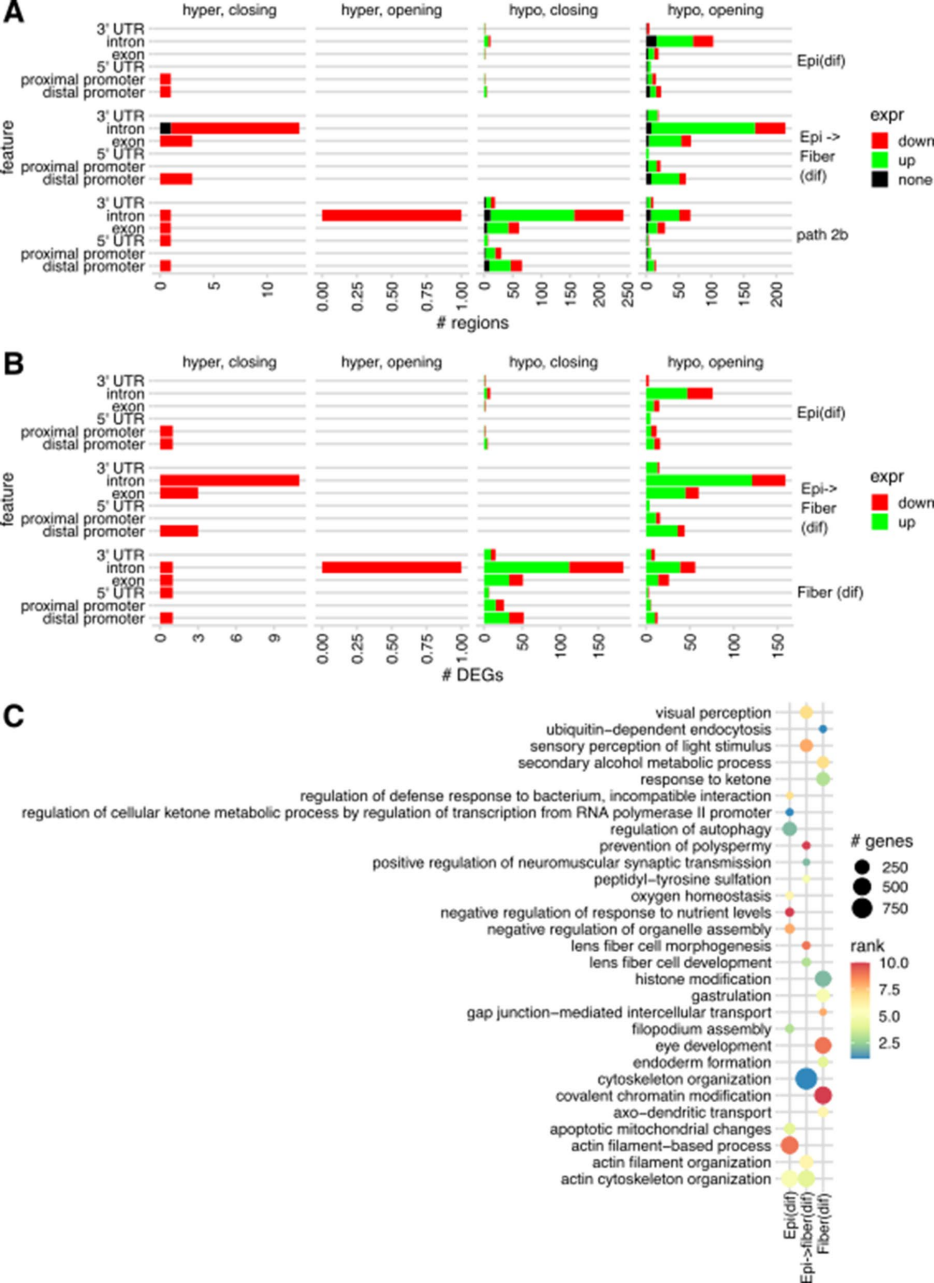
Differential DNA methylation, chromatin accessibility, and gene expression between in lens differentiation paths Epi(dif), EpiFiber(dif), and Fiber(dif). **A** Numbers of differentially methylated and accessible regions (DMARs) associated with DEGs for each path segment. **B** Numbers of DEGs associated with differentially methylated and accessible regions for each path segment. **C** Enriched GO terms of differentially methylated and accessible regions. Figure shows top 10 biological processes from each path segment

To investigate whether the observed epigenetic signals of DNA methylation and chromatin accessibility propagated to gene expression, we investigated how they related to mRNA levels. We determined differentially expressed genes (DEGs) for each of the three developmental path segments (see Fig. 1C) from a previously published RNA-seq dataset [43]. We then annotated regions where DMRs and DARs intersected, calling these regions as differentially methylated and accessible regions (DMARs). We particularly examined the DMARs associated with DEGs. We found that most DMARs were indeed associated with DEGs across all three paths. The majority of such regions were found in introns, followed by 1–5 kb upstream regions. The majority of DEG-associated DMARs were hypomethylated in accordance with the overall DMR population. Interestingly, for path Fiber(dif), hypomethylated DMARs appeared to be more frequently associated with closing chromatin. In contrast, in both paths Epi(dif) and EpiFiber(dif), the combination of demethylation and opening chromatin prevailed over the opposite trend (Fig. 10A).

Counting DEGs associated with DMARs, we found that hypomethylated-opening DMARs across all three paths were associated with hundreds of mostly upregulated DEGs. Notably, the hypomethylated-closing DMARs in path Fiber(dif) were associated with a similar distribution of upregulated and downregulated DEGs as hypomethylated-opening DMARs across all three paths (Fig. 10B). GO analysis of DMARs revealed multiple high-ranking eye-specific biological process terms, i.e., lens fiber cell morphogenesis and lens fiber cell development in path EpiFiber(dif) and “eye development” in path Fiber(dif) (Fig. 10C).

### DNA methylation and Pax6 binding

#### Global analysis of DNA methylation, open chromatin and Pax6-binding in lens chromatin

**Fig. 11:**
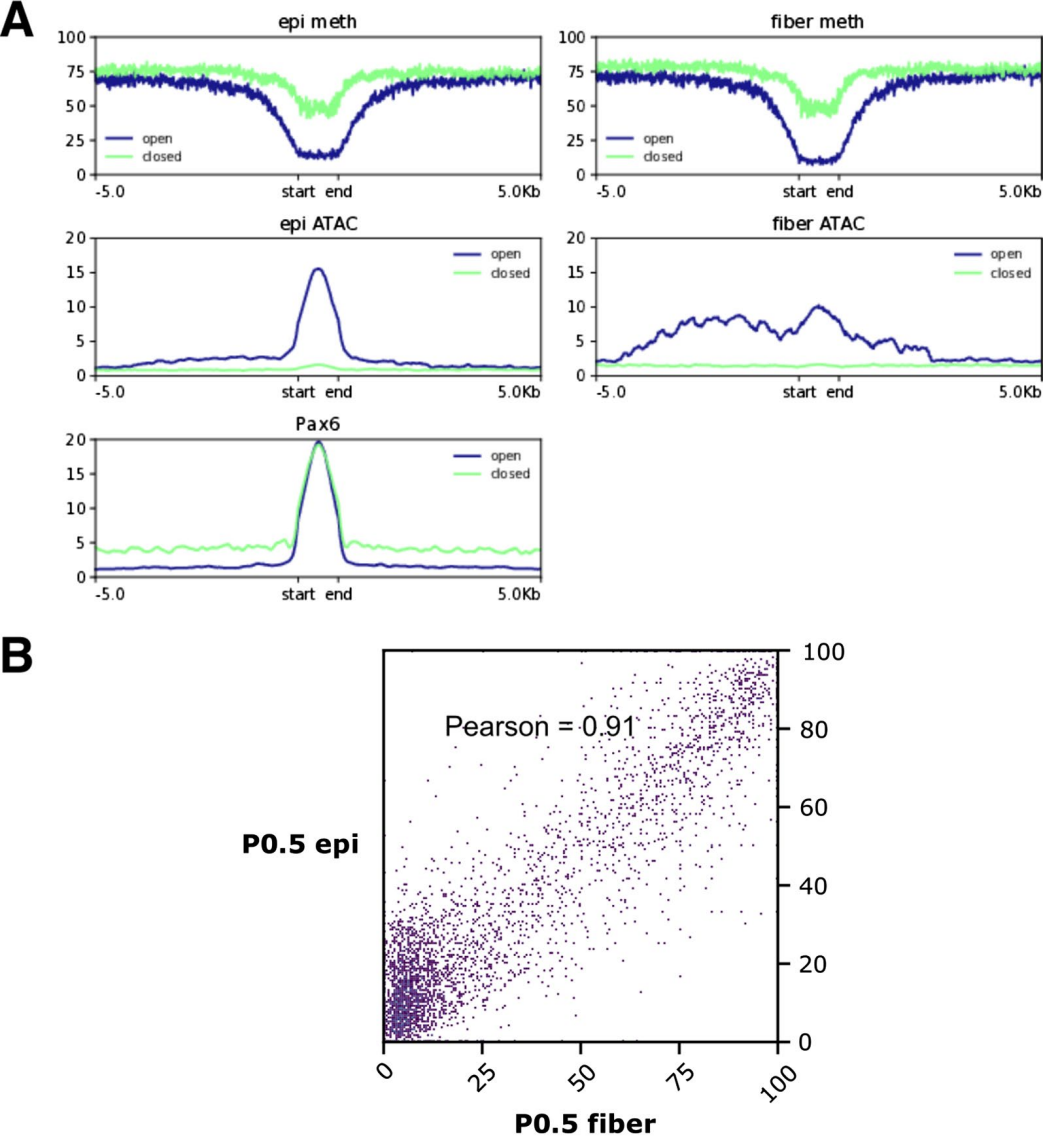
DNA Methylation and chromatin accessibility in Pax6 ChIP-seq peaks. **A** Profiles of DNA methylation in lens samples and chromatin accessibility via ATAC-seq in Pax6 peaks in newborn, sorted by regions of open chromatin in either epithelium or fiber and closed chromatin in both lens samples. Pax6 peaks appear in both open and closed chromatin, with the center of open chromatin regions showing demethylation overall. **B** Mean methylation within Pax6 peaks in representative epithelium and fiber samples. DNA Methylation within Pax6 peaks in the whole lens strongly correlated between epithelium and fiber. While Pax6 peaks concentrate towards regions showing low methylation in both epithelium and fiber, a significant number of peaks occupy regions with high methylation in both cell types

Pax6 is a major regulator of gene expression in the developing embryonic lens [86]. We thus investigated global relationship between DNA methylation and Pax6 DNA-binding in newborn lens through cross-analysis of WGBS and existing comparable ATAC-seq datasets as well as whole-newborn lens Pax6 ChIP-seq data [31]. We classified Pax6 peaks from whole-lens ChIP-seq into two types: associated with open or closed chromatin based on whether they intersected the merged ATAC-seq peaks from newborn epithelium and fibers (Fig. 11A). DNA methylation was low in the center of open chromatin-associated Pax6 peaks; however, this decrease was either absent or weaker in closed chromatin-associated Pax6 peaks, suggesting that demethylation promotes Pax6 binding only in open chromatin (Fig. 11A). Chromatin accessibility is high near the center of Pax6 peaks for both newborn epithelium and fiber; however, in fiber, surrounding regions within 5 kb of called peak boundaries also show high accessibility (Fig. 11A). The methylation levels in newborn epithelium and fiber within whole-lens Pax6 peaks are highly correlated, showing that methylation is unlikely to drive differences in Pax6 binding between cell types (Fig. 11B).

Finally, we noted that a majority of whole-lens Pax6 peaks in newborn lens did not intersect with any DNA-demethylated regions from either epithelium or fiber, suggesting that demethylation is not required for Pax6 binding. Of the Pax6 peaks that intersected with demethylated regions, most were associated with regions common to epithelium and fiber (Table 4).

**Table 4:** Pax6 peaks across genomic features and intersections with UMRs/LMRs in newborn mouse

Lens compartment of UMR or LMR	Distal promoter	Proximal promoter	5′ UTR	Exon	Intron	3′ UTR	Intergenic
Epithelium	12	6	1	11	47	1	34
Fiber	10	5	0	9	83	4	54
Shared	158	182	94	152	372	12	160
No UMR or LMR	169	67	21	147	1439	36	1173

Counts of intersections between Pax6 ChIP-seq peaks and UMRs/LMRs sorted by cell specificity in newborn mouse

#### In vivo* analysis of Pax6 binding and DNA methylation at Pax6 and Prox1 loci*

**Fig. 12:**
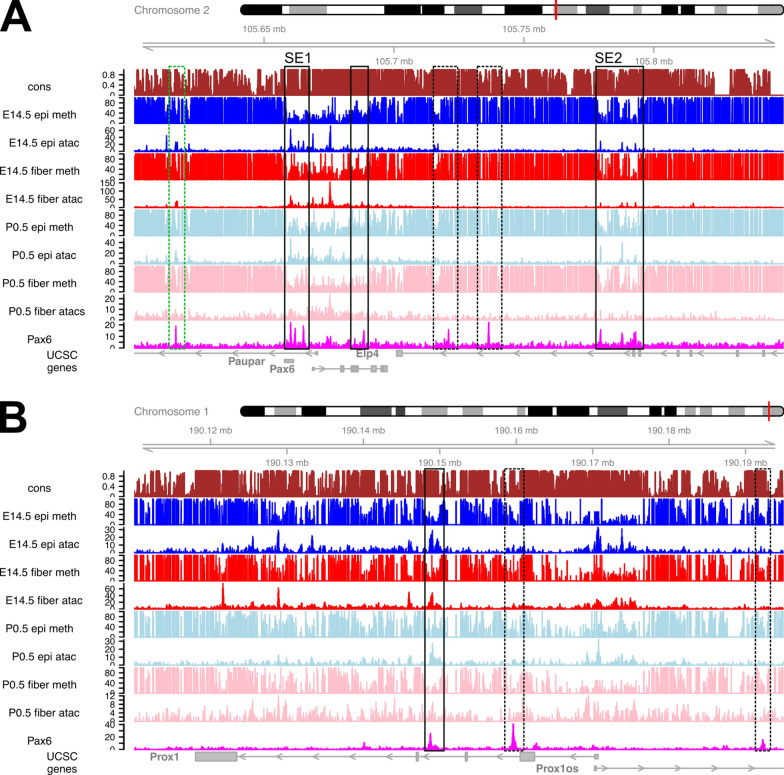
DNA methylation, chromatin accessibility and Pax6-binding at the *Pax6* and *Prox1* loci in lens chromatin. **A** *Pax6* locus, including *Paupar* (*Pax6os1*) and portion of *Elp4*. Two super-enhancer regions SE1 and SE2 are marked. Three regions of Pax6 binding, reduced methylation and open chromatin (boxed), 5′-distal region of Pax6 binding, high methylation and open chromatin of unknown function (green box), two regions of Pax6 binding, high methylation and closed chromatin (dashed boxes). **B** *Prox1* locus. A region of Pax6 binding, reduced methylation and open chromatin (boxed), two regions of Pax6 binding, mostly closed chromatin and presence of DNA methylation (dashed boxes). The individual tracks include evolutionary conservation (cons) and DNA methylation (see Fig. 1), ATAC-seq (see Fig. 9) and Pax6-binding (newborn whole lens chromatin)

The Pax6 binding sites were determined earlier by ChIP-seq in newborn (P0.5) lens chromatin [31]. Subsequently, these sites were found both in open and closed chromatin domains [44]. To visualize individual trends in Pax6-binding and DNA methylation described above (Fig. 11A), we focused on two loci, *Pax6* and *Prox1* (Fig. 12) as each of them contains multiple Pax6-bound regions [31]. Importantly, Pax6 locus autoregulates itself using multiple distal enhancers [86–88] and Prox1 is a direct Pax6 target gene in the lens [31]. Six Pax6-bound regions within the > 400 kb genomic region of lens chromatin are marked by boxes and some of them contain multiple Pax6 peaks (Fig. 11A). Four of these regions (boxes) are located in open lens chromatin, particularly super-enhancers SE1 and SE2, containing well-characterized individual 5′-EE [89–92] and 3′-SIMO distal enhancers [88, 89, 93], respectively. LMR/UMR are found within SE1 and SE2 [31]. Likewise, the most distal 5′-located Pax6 binding region (green box) with unknown function is also located in an “open” chromatin region. In contrast, two Pax6 peaks (dotted boxes), located in the 3′region (last intron of the *Elp4* locus) are located in both closed and methylated lens chromatin (Fig. 12A).

Three Pax6 peaks were found in the ~ 80 kb *Prox1* locus (Fig. 12B). The most 3′-distal peak (black box) is found in open chromatin in each lens sample and shows the lowest levels of DNA methylation correlating with higher expression of Prox1 in lens fibers compared to lens epithelium [94, 95]. Another 3′-distal region contains “stronger” Pax6 peak; however, its surrounding chromatin has lower “open” chromatin signal (dashed box). The 5′-upstream Pax6 peak (dashed box) is in open chromatin only in P0.5 lens fibers and the region shows higher methylation compared to the other two Pax6 peaks located in the evolutionarily conserved intronic *Prox1* regions. Taken together, these two examples show that Pax6-binding to chromatin in vivo can occur in both UMRs, LMRs and methylated regions of two genes, Pax6 and Prox1, known to be directly regulated by this transcription factor [31].

#### In vitro* analysis of Pax6 binding to normal and cytosine-methylated sites*

**Fig. 13:**
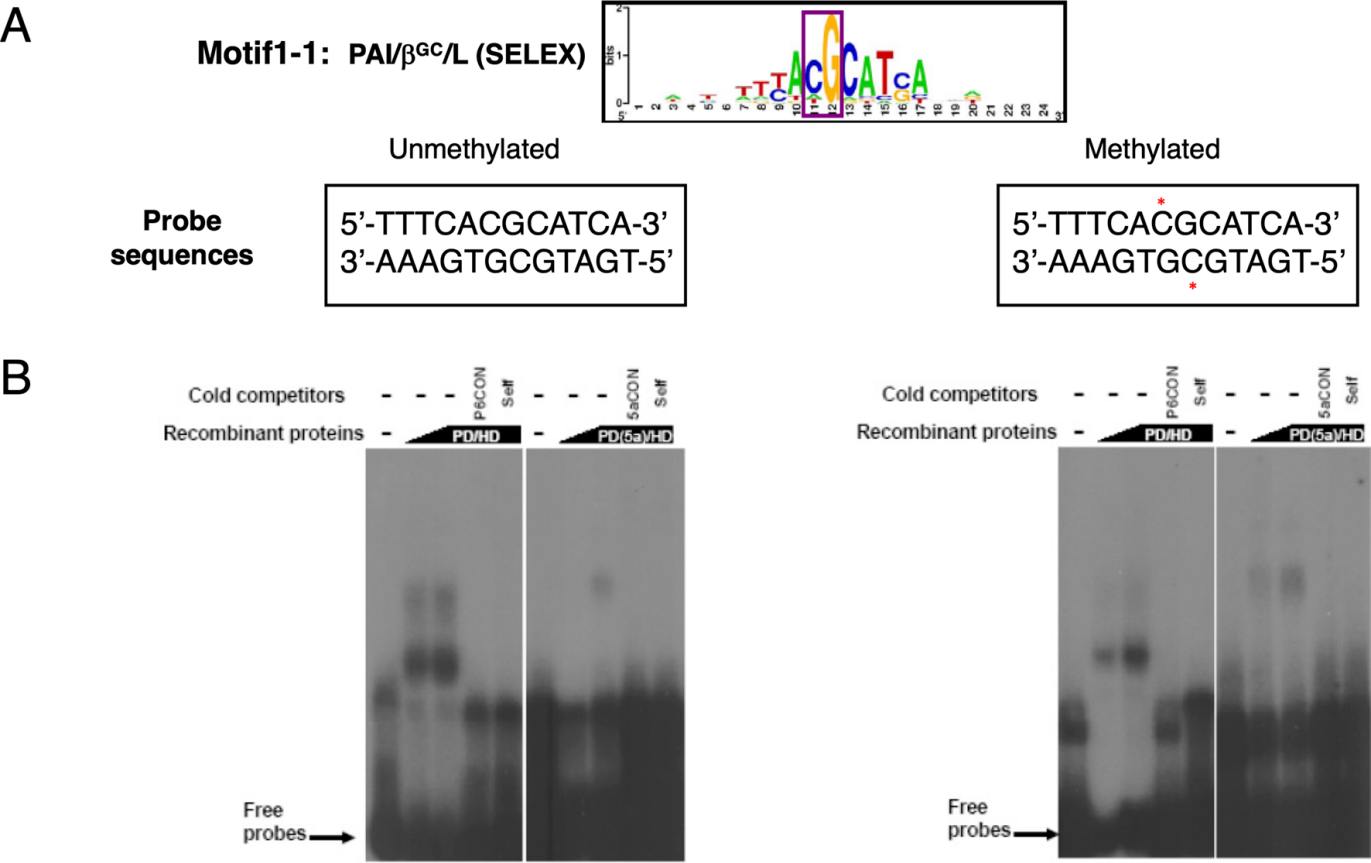
In vitro Pax6 binding to sites with a single unmethylated and methylated CpG dinucleotide. **A** The consensus motif 1–1 [86] is shown followed by individual binding sites with C residues methylated marked by asterisks (red). **B** EMSA results using Pax6 PD/HD and PD(5a)/HD proteins. The specificity of the complexes is demonstrated by competition with cold oligonucleotides containing consensus Pax6 binding sites (P6CON) and unlabeled self-oligonucleotide. The autoradiography of experiments with PD/HD and PD(5a)/HD proteins were 6 and 16 h, respectively

**Fig. 14:**
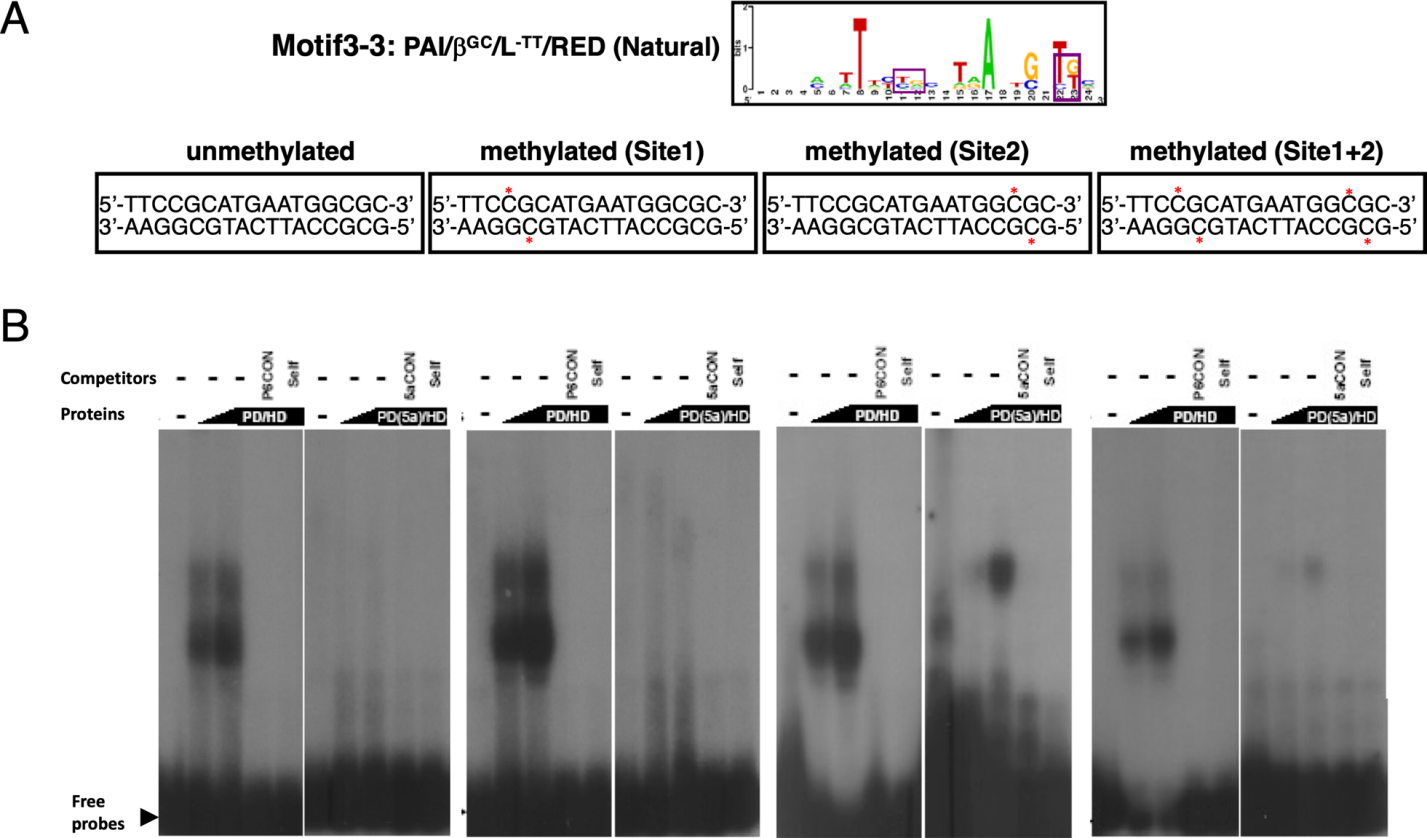
In vitro Pax6 binding to sites with two unmethylated and methylated CpG dinucleotides. **A** The consensus motif 3–3 [86] is shown followed by individual binding sites with two or four C residues methylated marked by asterisks (red). **B** EMSA results using Pax6 PD/HD and PD(5a)/HD proteins. The specificity of the complexes is demonstrated by competition with cold oligonucleotides containing consensus Pax6 binding sites (P6CON) and unlabeled self-oligonucleotide. The autoradiography of experiments with PD/HD and PD(5a)/HD proteins were 6 and 16 h, respectively.

Earlier studies of Pax6 binding revealed presence of one [86, 96] or two [86] CpG dinucleotides in optimal Pax6-binding sites generated in vitro by SELEX and through an alignment of experimentally validated Pax6-binding sites. Here we used two DNA-binding sequences with optimal binding sites for both Pax6 paired domain (PD) and homeodomain (HD) mostly recognized by the N-terminal PAI-subdomain (motif 1-1) and recognized by both PAI and RED subdomains (motif 3-3) [86] (see Figs. 13 and 14). The motif 1-1 oligonucleotides, either unmethylated or methylated, were incubated with recombinant Pax6 proteins containing PD/HD and PD(5a)/HD and analyzed by electrophoretic mobility shift assays (EMSAs) (Fig. 13). Both unmethylated and methylated probes bound Pax6 proteins although the individual complex band intensities varied.

The patterns found with motif 3-3 were more complex (Fig. 14). Methylation of the upstream CpG slightly increased PD/HD binding while the minor form of Pax6, PD(5a)/HD, did not bind both probes as shown for the unmethylated probe earlier [97]. Methylation of the downstream CpG had a very modest positive effect; however, this probe also bound PD(5a)/HD proteins. Methylation of both CpGs reduced binding of PD/HD and PD(5a)/HD proteins compared to the downstream mono-methylated site (Fig. 14). Taken together, these in vitro studies agree with in vivo Pax6 binding found in methylated genomic regions.

### Comparison of regulatory mechanisms between E14.5 embryonic lens epithelium and newborn P0.5 lens fibers

**Fig. 15:**
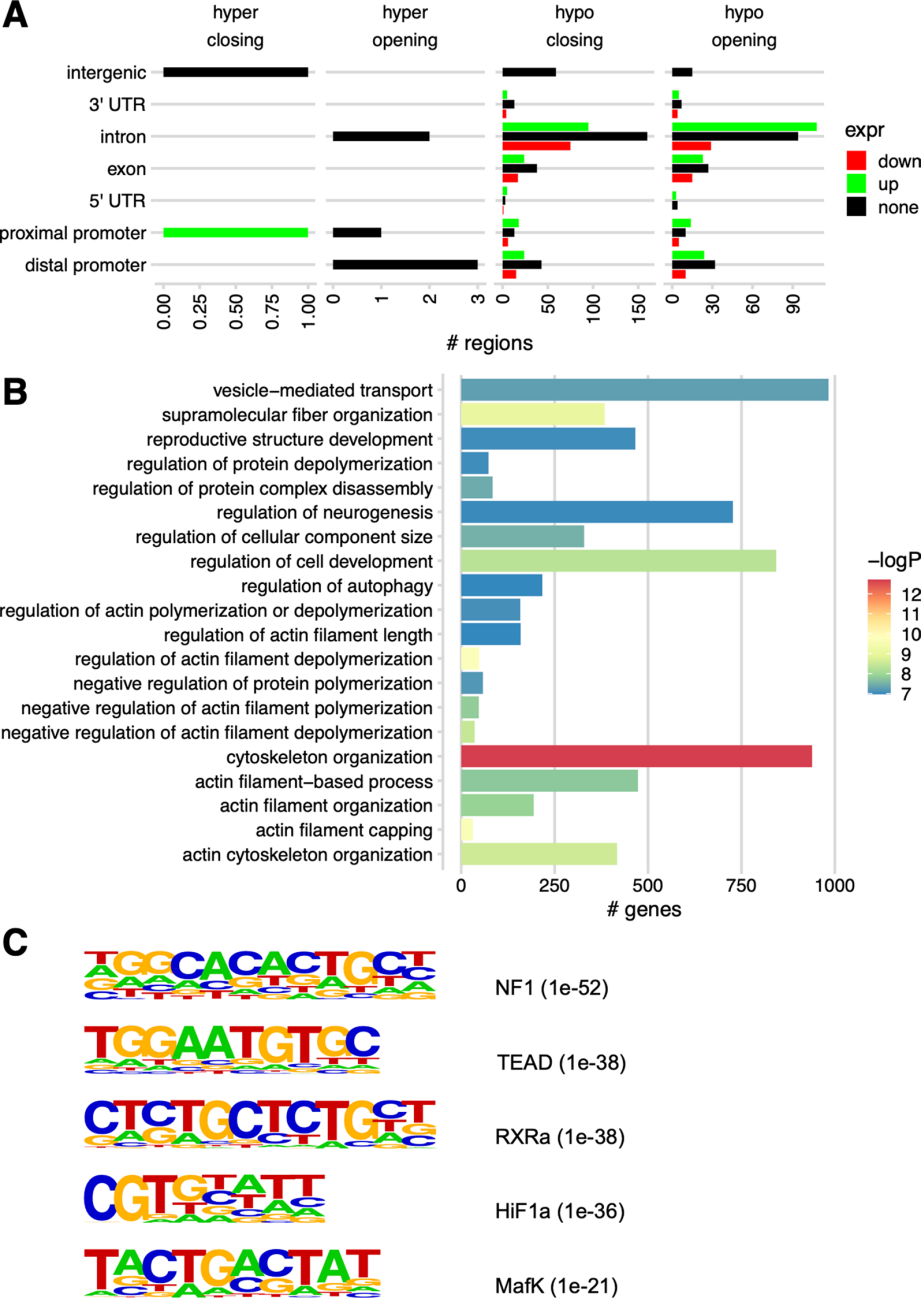
Direct comparison of differential methylation and chromatin accessibility between E14.5 embryonic epithelium and newborn P0.5 fibers. **A** Numbers of differentially methylated and accessible regions and their associations with DEGs. **B** The top 20 gene ontology terms obtained from differentially methylated-accessible regions. **C** Five selected de novo enriched motifs in this differentiation path, hypomethylated DMRs, best match results for transcription factors recognizing these motifs, and enrichment p-values (brackets). For the full results of the de novo enriched motif analysis, see Additional file 9: Table S7. No significantly enriched de novo motifs were found for hypermethylated DMRs

To determine the genes and functions most impacted by epigenetic regulation over time in developing lens, we performed a direct comparison of methylation between E14.5 embryonic epithelium and P0.5 newborn lens fibers, effectively integrating analyses performed on developmental paths EpiFiber(dif) and Fiber(dif). We compared genomic regions that showed unidirectional changes (i.e., continuously increased or decreased) in DNA methylation and chromatin accessibility with genes that showed unidirectional changes in mRNA expression during differentiation from embryonic epithelium to newborn fiber. We found hundreds of DMARs containing hypomethylated DMRs and both opening and closing DARs associated with DEGs; most such regions were found in gene introns and 1–5 kb upstream promoter regions. A comparable number of such regions with similar distribution across genomic features were associated with genes that showed no change in expression, or with intergenic regions (Fig. 15A). For the full list of such regions, see Additional file 8. GO analysis of these regions found the most significance in biological process terms actin regulation and cytoskeleton organization (Fig. 15B), both well known to control extensive elongation of lens fiber cell cytoskeleton [36, 98].

To infer transcription factors whose activity may be affected by differential methylation during this developmental process, we performed motif enrichment analysis on DMRs between E14.5 epithelium and P0.5 fibers. This analysis revealed significantly enriched sequences with high similarity to known binding motifs for NF1, TEAD, RXRα, Hif1α, and MafK in hypomethylated DMRs between E14.5 epithelium and P0.5 fiber (Fig. 14C). For the full result of the motif analysis, see Additional file 9. Importantly, previous studies have shown major roles of c-Maf [99–101], Hif1α [102–104], MafG and MafK [105, 106], and RAR/RXR [107–109] in control of lens-specific transcription. The NF1 and TEAD motifs were also previously identified using open chromatin analysis [44]; nevertheless, no data are available for individual transcription factors recognizing these motifs from previous lens studies. Taken together, these unbiased chromatin and DNA methylation patterns leading to the above *cis*-regulatory grammar and individual transcription factors are supported by early functional studies of multiple DNA-binding transcription factors during lens development.

## Discussion

The present studies demonstrate major differences in DNA methylation patterns between lens cells and other cell types as well as gene-specific increase of unmethylated regions in both differentiating lens fibers and lens epithelial cells. Despite the presence of CpG dinucleotides in Pax6-binding sites, both in vivo and in vitro data show that in general DNA methylation does not obstruct binding of Pax6 proteins to DNA. These lens data can also serve for comparative purposes of mouse organogenesis studies, between mammalian and avian models [47], and between DNA isolated from human normal and cataract lenses [53, 54, 110].

Earlier genetic studies on DNA methylation during lens development are limited to conditional inactivation of *Dnmt1*, *Dnmt3a* and *Dnmt3b* [57] as no similar data are available for *Tet1*, *Tet2* and *Tet3* genes. In addition, expression of de novo DNA methyltransferases dnmt3–dnmt8 is available for zebrafish lens [111]. Here, we show RNA-seq data on these genes to understand their expression dynamics during lens differentiation (Additional file 10). In general, these data show higher expression levels of Dnmt1, Dnmt3b, Tet1 and Tet2 in lens epithelial cells compared to fibers. Both Dnmt3a and Tet3 are expressed at comparable levels in both lens compartments. Expression of epigenetic integrator Uhrf1 [112] is shown for a comparison. Thus, the demethylation events in lens fibers most likely reflect functions of the Tet3 enzyme. However, the challenge for these loss-of-function studies during lens development is the lack of ideal cre-lines for conditional gene inactivation of these global regulators of epigenetic processes. The results of the available studies [57] suggest that the critical events that control lens methylome occur during the formation of lens progenitors and precursor cells, no later than between E9.5 to E11.5 of mouse embryonic development.

The present data mapped histone variant H3.3 localization in chromatin prepared from newborn lens epithelium and lens fibers. As expected, crystallin loci show marked presence of H3.3 (Fig. 9) as these genes are expressed at very high levels directly comparable to globin genes in erythrocytes [79]. An increase of H3.3 signals is notable in postmitotic terminally differentiating lens fibers (Fig. 8B). Note strong localization of RNA polymerase II has been shown across crystallin gene coding regions [79]. Our ongoing studies are aimed to map both single and multiple posttranslational modifications of individual histone H3.1, H3.2 and H3.3 variants located on the same histone tail using comparative unbiased proteomic analyses [113].

The present data show aggregated multi-omics data on several representative genes, including crystallins (Fig. 9) and lens-regulatory transcription factors Pax6 (Figs. 3 and 12A), Prox1 (Fig. 12B), Foxe3, gap junction proteins, and MIP/aquaporin0 (Additional file 7). In addition, lens methylation profiles at cataract-related loci include Chmp4b, Col4a1–Col4a2, and Lss (Additional file 11) may help studies of aging human lenses and formation of cataracts [114–119].

Earlier studies have shown that binding of specific DNA-binding transcription factors is modulated by their cytosine methylations [7] also affecting local DNA shape [120]. Related to eye development, the examples of methylation-sensitive transcription factors include AP-2α, Crx, Gata3, Rbpj, RXRα, and Smad4 [7]. Pax6 is a PD and HD-containing transcription factor that plays multiple critical roles in brain, eye and pancreas development [32, 33, 65, 69–72]. Our previous studies in the lens and forebrain identified in vivo DNA-binding motifs [31] that are consistent with earlier SELEX-driven assays [86, 96]. Interestingly, the most common Pax6-binding motif recognized by the N-terminal PAI-subdomain contains a single CpG, while the other variant site mostly recognized by the C-terminal RED subdomain contains two CpG dinucleotides separated by 10 bps [86]. Given that binding of Pax6 in vivo is both in open and closed lens and forebrain chromatins [44]; it is of general interest to link DNA methylation with Pax6 occupancy to advance of our understanding of gene control during lens differentiation. Our data demonstrate in vivo binding of Pax6 in both low-methylated and methylated chromatin lens domains. Thus, DNA changes caused by CpG methylation [120] are not involved in regulation of Pax6-binding, and this is consistent with critical roles of Pax6 in establishment of novel cell lineages, such as the lens [33, 121] and key roles of Pax6 in eye evolution [122, 123].

Although the expression of Pax6 determined by transcriptomic studies and amount of Pax6 proteins is lower in lens fibers compared to lens epithelium [124, 125], genetic loss-of-function studies clearly demonstrate that Pax6 regulates lens fiber cell differentiation [126]. To further probe the roles of DNA-methylation, Pax6-binding to DNA and role of Pax6 in epigenetic regulation of cell development, ChIP-seq studies of Pax6 and other lens-regulatory transcription factors must be conducted using microdissected mouse lenses. Importantly, mouse lens data already exist that functionally link Pax6 with various chromatin remodeling complexes, including BAF complexes with Brg1 (Smarca4) [127–129], ISWI complexes including Snf2h (Smarca5) [129, 130], Mll/Set1 complexes [129] and p300/CBP [129–132] and generation of open chromatin [44].

Future studies will be required to implement multi-omics approaches at single cell levels [4–6] in mid-stages of mouse embryogenesis to define methylomes of early lens progenitor cells and compare them with naïve ectoderm and primitive neuroectoderm. In vitro differentiation of Tet-depleted and control mouse ES cells into lens progenitors provides another attractive approach to probe changes in DNA methylation during cell fate decisions [19], including lens cell formation.

The current data are useful for comparative studies between mouse and chicken lens development. The available chick data from E13 embryos (HH stage 39) [47] represent more advanced stage of secondary fiber cell differentiation as the primary lens fiber cells are generated already in E4.5 embryos and are rather comparable to E13.5–E14.5 mouse embryos. Regarding the crystallin gene expression, the major difference between these models is that the birds recruited δ1-crystallin/argininosuccinate lyase as their major crystallin gene [133, 134] and MafA/L-Maf (chick chromosome 2) was implicated as the important transcription factor regulating crystallin gene expression in the avian lens [135] while mouse MafA null lenses appear normal [136]. In mice, a structurally similar c-Maf regulates crystallin gene expression [99–101] and its chick homologue is located on chromosome 11. The present mouse data (Fig. 9B) show variable methylation in the bi-directional promoter region of the *Crybb1–Cryba4* loci [81]. The chicken data from a syntenic region also revealed similar patterns with more profound reduction of DNA methylation in fiber cell chromatin within the *CRYBB1* promoter compared to the adjacent *CRYBA4* promoter [47]. Both chicken and mouse data facilitate comparative analyses of chromatin landscape and prioritization of candidate distal enhancers for their functional studies such as those already identified in the chicken *SOX2* locus [60–63].

In conclusion, the present study has generated the first data on methylation changes between two different stages of mammalian lens development. Analysis of histone H3.3 variant was also performed in microdissected newborn lenses. Comprehensive analysis of these data included chromatin accessibility maps generated by ATAC-seq, gene expression data by RNA-seq, and Pax6 binding by ChIP-seq in newborn whole lens. Both the present mouse and earlier chicken studies [47] demonstrate that reduced DNA methylation correlates with expression of important genes involved in lens morphogenesis and lens fiber cell differentiation, including genes encoding crystallins, intermediate filament proteins, and lens fiber cell membrane proteins. In addition, a number of genes subjected to the DNA methylation control encode proteins playing multiple roles in general cellular process such as cytoskeleton organization, covalent chromatin modifications, regulation of autophagy, negative regulation of organelle assembly, and gap junction-mediated intercellular transport, that provide rich resource for their functional studies using diverse model organisms.

## Materials and methods

### Tissue samples and WGBS

Mouse lenses from E14.5 and P0.5 CD1 mice (Charles River Laboratories) were microdissected into epithelium and fibers under the microscope as we described earlier [43, 44, 137]. Ten P0.5 and thirty E14.5 lenses were used per sample. Three biological replicates were obtained. Genomic DNA was isolated from all samples using QIAamp DNA Mini Kits (Cat. ID 51304) followed by isopropanol precipitation (− 80 °C, 1 h incubation). The genomic DNA samples were then used for WGBS library construction. The WGBS libraries were generated at the New York Genome Center, as we previously reported [138].

### Lens WGBS data analysis and external DNA methylation data

Lens WGBS reads were mapped to the GRCm38/mm10 mouse genome and the methylation at individual CpGs scored using bismark 0.18.1 [139] and bowtie 2.3.3.1 [140]. Annotation of sampled CpGs was performed with annotatr [141]. The genome annotation includes intergenic, 1–5 kb upstream regions (labeled in this text as distal promoter), promoter (labeled in this text as proximal promoter), 5′ untranslated region (UTR), exon, intron and 3′ UTR DNA sequences. The DNA methylation data for ES cells (GSE82125) and NPCs [58] were used for comparative analyses.

### Identification and annotation of demethylated and DMRs

Methylation scores for lens samples were smoothed and DMRs called using the R package bsseq 1.26.0 [142]. Demethylated regions were called on the bsseq-smoothed methylation scores using the R package MethylSeekR 1.30.0 [143]. Annotations associating genomic regions with genes, genomic features, and intergenic regions were performed using the R package annotator 1.16.0 [141]. For these annotations, note that a single region can intersect multiple different features within the same gene (e.g., distal and proximal promoters); in such cases, we chose to count the region once for each genomic feature with which it was associated. GO analysis of demethylated regions, differentially methylated regions, and other genomic regions was performed using GREAT 4.0.4 [144]. For full output of these analyses, see Additional file 2: Tables S2, Additional file 4: Table S4.

### Integration of WGBS data with prior ATAC-seq, RNA-seq, and ChIP-seq data

Processed ATAC-seq data and DARs were obtained from [44]. RNA-seq data were obtained from the count matrix from [43]. DEGs were calculated with DESeq2 [145] using default settings. ChIP-seq data were obtained from previous work [31]. Reads from the prior lens ChIP-seq dataset originally mapped to the mm9 genome were re-mapped to the mm10 genome using bowtie2 version 2.2.3. ChIP-seq peaks [31] originally called with the mm9 genome were lifted to mm10 using liftOver [146]. Intersections between demethylated regions or DMRs and ChIP-seq or ATAC-seq peaks were determined using bedtools 2.30.0 [147].

For the integration of DMR information with chromatin state shown in Table 3, we defined for each path stable open chromatin as the intersection of all ATAC-seq peaks not intersecting DARs. Similarly, we defined stable closed chromatin as the complement of stable open chromatin regions not intersecting DARs. We used bedtools 2.30.0 to determine each group of regions and obtain their intersections with DMRs.

### ChIP-seq analysis of histone H3.3

Two hundred microdissected P0.5 lenses were obtained from CD-1 mice (Charles River Laboratories) and stored in liquid nitrogen prior the use as we described earlier [31]. Preparation for ChIP-seq was provided by ActiveMotif (Carlsbad, CA, USA). In brief, immunoprecipitation was performed on 12 µg chromatin from microdissected lens cells with 5 µl anti-H3.3 antibody (Millipore, cat. # 17-10245), *n* = 2 biological replicates, at ActiveMotif. The 75-nt single-end (SE75) sequence reads generated by Illumina sequencing (using NextSeq 500) are mapped to the genome using the BWA algorithm (“bwaaln/samse” with default settings) [148]. Peaks were called using SICER2 [149]. Intersections between histone H3.3 peaks and DMRs were found using bedtools 2.30.0. Gene ontology analysis was performed with GREAT [144].

### EMSAs

The GST-fusion Pax6 PD/HD and PD(5a)/HD proteins were expressed in *E. coli* and isolated as we described elsewhere [86]. Two different Pax6-binding gel-purified oligonucleotides (binding regions, underlined), 5′-GAAAACGAGTATTCACGCATCACAAAACAAAGAGCT-3′ (motif 1–1) and 5′-TTCAGGAAAATTTCCGCATGAATGGCGCAGCTCGAGT-3′ (motif 3–3) were used and their individual or both CpG residues were symmetrically methylated during their initial synthesis (Fisher Scientific). The EMSAs were performed as we described elsewhere [86, 150].

### Motif analysis of lens differentiation path Epi(E14.5) to Fib (P0.5) and its associated DMRs

Motif analysis was performed with HOMER 4.7 [151] using the -noweight option to disable normalization for GC content. Results from enrichment of de novo motifs were considered.

### R version and data visualization

All R analyses were performed using R version 4.0.3. Graphics were made in R using packages cowplot 1.1.1 and ggplot2 3.3.5 [152]. Heatmaps and profile plots were made with deeptools 3.5.1 [153].

## Supplementary Information

**Additional file 1: Table S1.** Genic annotations, binomial rank, and binomial p-values of UMRs and LMRs associated with GO biological process terms shown in Fig. 4A. Mean RPKM of associated gene (data from [43]).

**Additional file 2: Table S2.** Complete GREAT gene ontology analysis output of reproducible demethylated regions in lens cells.

**Additional file 3: Table S3.** Complete GREAT gene ontology analysis output of DMRs.

**Additional file 4: Table S4.** Complete GREAT gene ontology analysis output of H3.3 peaks.

**Additional file 5: Table S5.** Annotation including associated gene and genomic feature of intersections between DMRs and histone H3.3 peaks.

**Additional file 6: Figure S1.** Mammalian conservation, lens methylation, lens ATAC-seq read density, lens H3.3 ChIP-seq read density, and lens RNA polymerase II ChIP-seq read density at the *Cryaa* locus and *Cryga-Cryge* five gene cluster. ATAC-seq data [44], ChIP-seq data [31].

**Additional file 7: Figure S2.** Mammalian conservation, lens methylation, lens ATAC-seq read density, lens histone H3.3 ChIP-seq read density, and lens RNA polymerase II ChIP-seq read density at the *Bfsp1*, *Bfsp2*, *Gja3*, *Gja8*, *Mip*, and *Foxe3* loci. DMRs shown in colored boxes and methylation change indicated by colored text.

**Additional file 8: Table S6.** Complete lists of differentially methylated-accessible regions associated with DEGs for each of Epi(dif), EpiFiber(dif), Fiber(dif) and Epi(E14.5)Fiber (P0.5)(dif) path showing genomic feature and direction of change of methylation, chromatin accessibility, and gene expression.

**Additional file 9: Table S7.** Complete output of HOMER de novo motif search of path Epi(E14.5)Fiber (P0.5)(dif) hypomethylated DMRs.

**Additional file 10: Figure S3.** Expression levels of for Dnmt1, Dnmt3a, Dnmt3b, Tet1, Tet2, Tet3 and Uhrf1 in eight microdissected lens samples (E14.5, E16.5, E18.5 and P0.5). RNA-seq data [43].

**Additional file 11: Figure S4.** Mammalian conservation, lens methylation, lens ATAC-seq read density, lens histone H3.3 ChIP-seq read density, and lens RNA polymerase II ChIP-seq read density at three cataract-related loci: *Chmp4b*, *Col4a1*-*Col4a2* and *Lss*. DMRs shown in colored boxes and methylation change indicated by colored text.

## Data Availability

WGBS and H3.3 ChIP-seq data were deposited into the Gene Expression Omnibus (GEO) with accession ID GSE213901 (https://www.ncbi.nlm.nih.gov/geo/query/acc.cgi?acc=GSE213901).

## Abbreviations

ATAC-seq: Assay for transposase-accessible chromatin with high-throughput sequencing
ChIP-seq: Chromatin immunoprecipitation followed by next generation sequencing
DARs: Differentially accessible regions
DEGs: Differentially expressed genes
DMRs: Differentially methylated regions
DMARs: Differentially methylated and accessible regions
EMSAs: Electrophoretic mobility shift assays
ES: Embryonic stem
FDR: False discovery rate
FC: Fold change
GO: Gene ontology
GRN: Gene regulatory network
HAT: Histone acetyltransferase
HD: Homeodomain
LMRs: Low methylated regions
NPCs: Neuronal progenitor cells
PD: Paired domain
PTM: Posttranslational modification
PCA: Principal component analysis
3′-UTR: 3′-Untranslated region
5′-UTR: 5′-Untranslated region
TF: Transcription factor
TSS: Transcription start site
UMRs: Unmethylated regions
WGBS: Whole genome bisulfite sequencing
