# Supplementary material for: Dynamic changes in whole genome DNA methylation, chromatin and gene expression during mouse lens differentiation
Source: Epigenetics Chromatin. 2023 Jan 25;16:4. doi: 10.1186/s13072-023-00478-7 (PMC9875507; doi:10.1186/s13072-023-00478-7)
Supplement: Supplementary file 7 — Additional file 7: Figure S2. Mammalian conservation, lens methylation, lens ATAC-seq read density, lens histone H3.3 ChIP-seq read density, and lens RNA polymerase II ChIP-seq read density at the Bfsp1, Bfsp2, Gja3, Gja8, Mip, and Foxe3 loci. DMRs shown in colored boxes and methylation change indicated by colored text. [file 13072_2023_478_MOESM7_ESM.pdf]

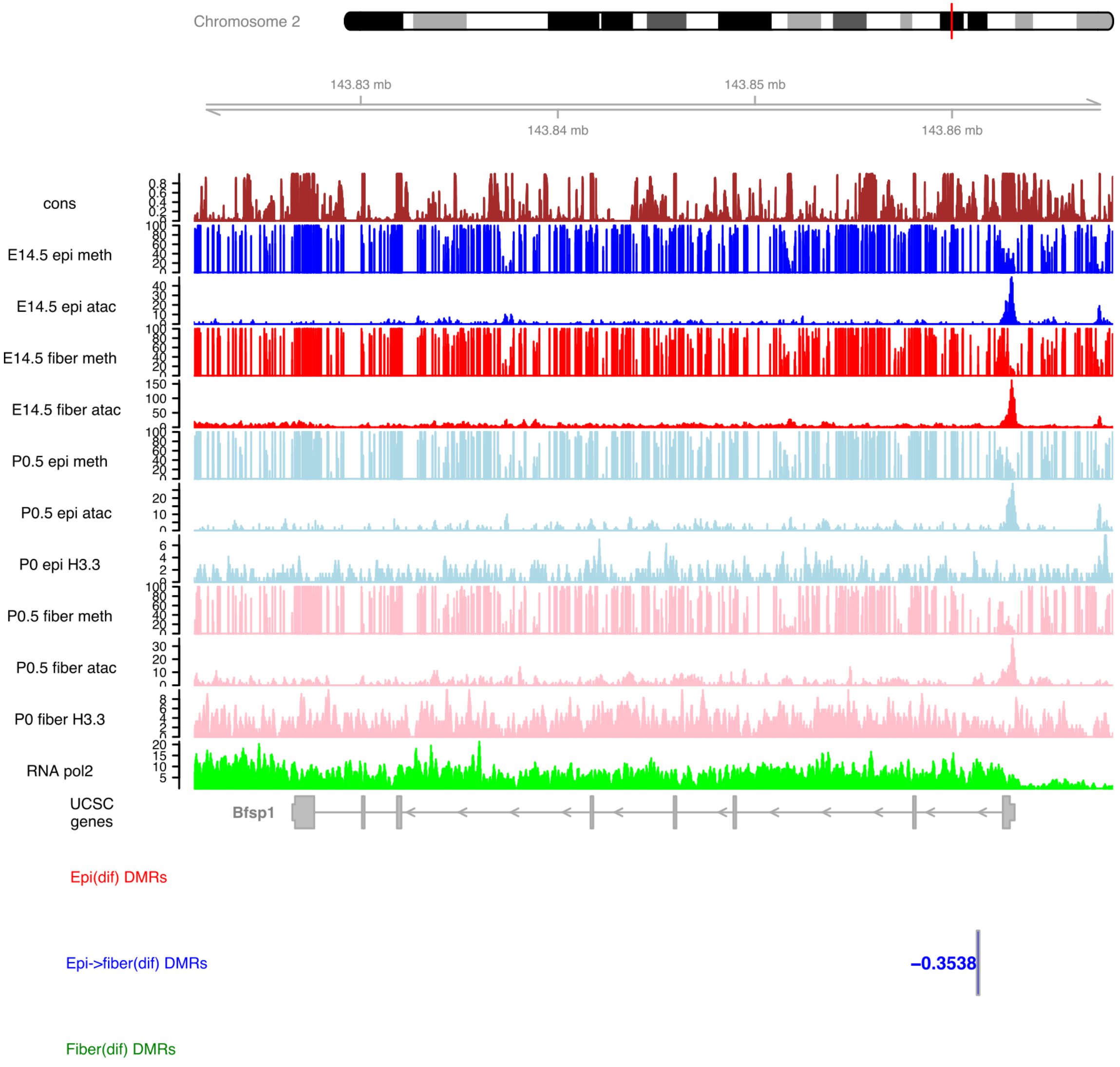
Chromosome 2
143.83 mb
143.85 mb
143.84 mb
143.86 mb
cons
E14.5 epi meth
E14.5 epi atac
E14.5 fiber meth
E14.5 fiber atac
P0.5 epi meth
P0.5 epi atac
P0 epi H3.3
P0.5 fiber meth
P0.5 fiber atac
P0 fiber H3.3
RNA pol2
UCSC genes
Bfsp1
Epi(dif) DMRs
Epi->fiber(dif) DMRs
−0.3538
Fiber(dif) DMRs

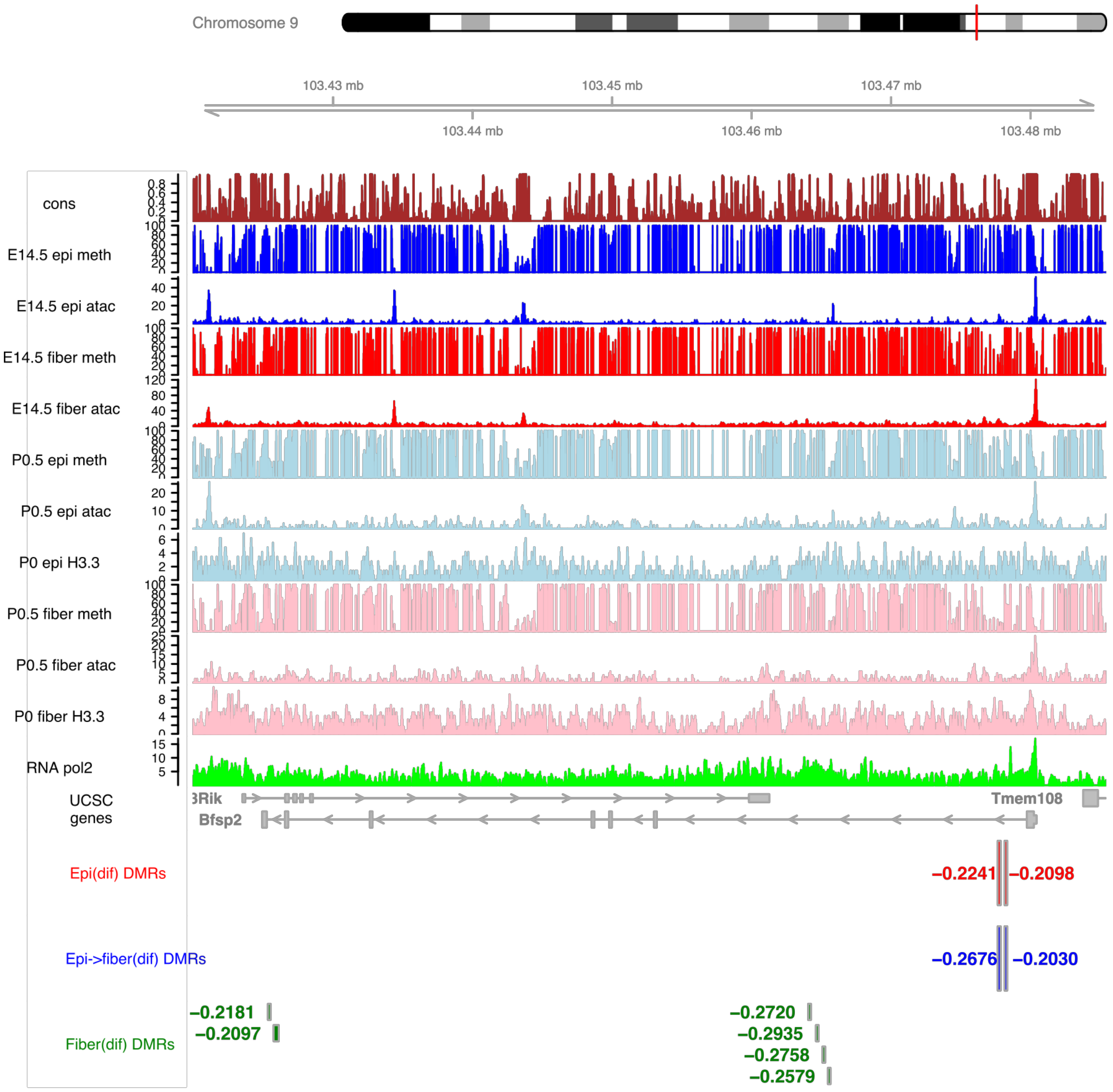
Chromosome 9
103.43 mb
103.45 mb
103.47 mb
103.44 mb
103.46 mb
103.48 mb
cons
E14.5 epi meth
E14.5 epi atac
E14.5 fiber meth
E14.5 fiber atac
P0.5 epi meth
P0.5 epi atac
P0 epi H3.3
P0.5 fiber meth
P0.5 fiber atac
P0 fiber H3.3
RNA pol2
UCSC genes
3Rik
Tmem108
Bfsp2
Epi(dif) DMRs
−0.2241
−0.2098
Epi->fiber(dif) DMRs
−0.2676
−0.2030
Fiber(dif) DMRs
−0.2181
−0.2097
−0.2720
−0.2935
−0.2758
−0.2579

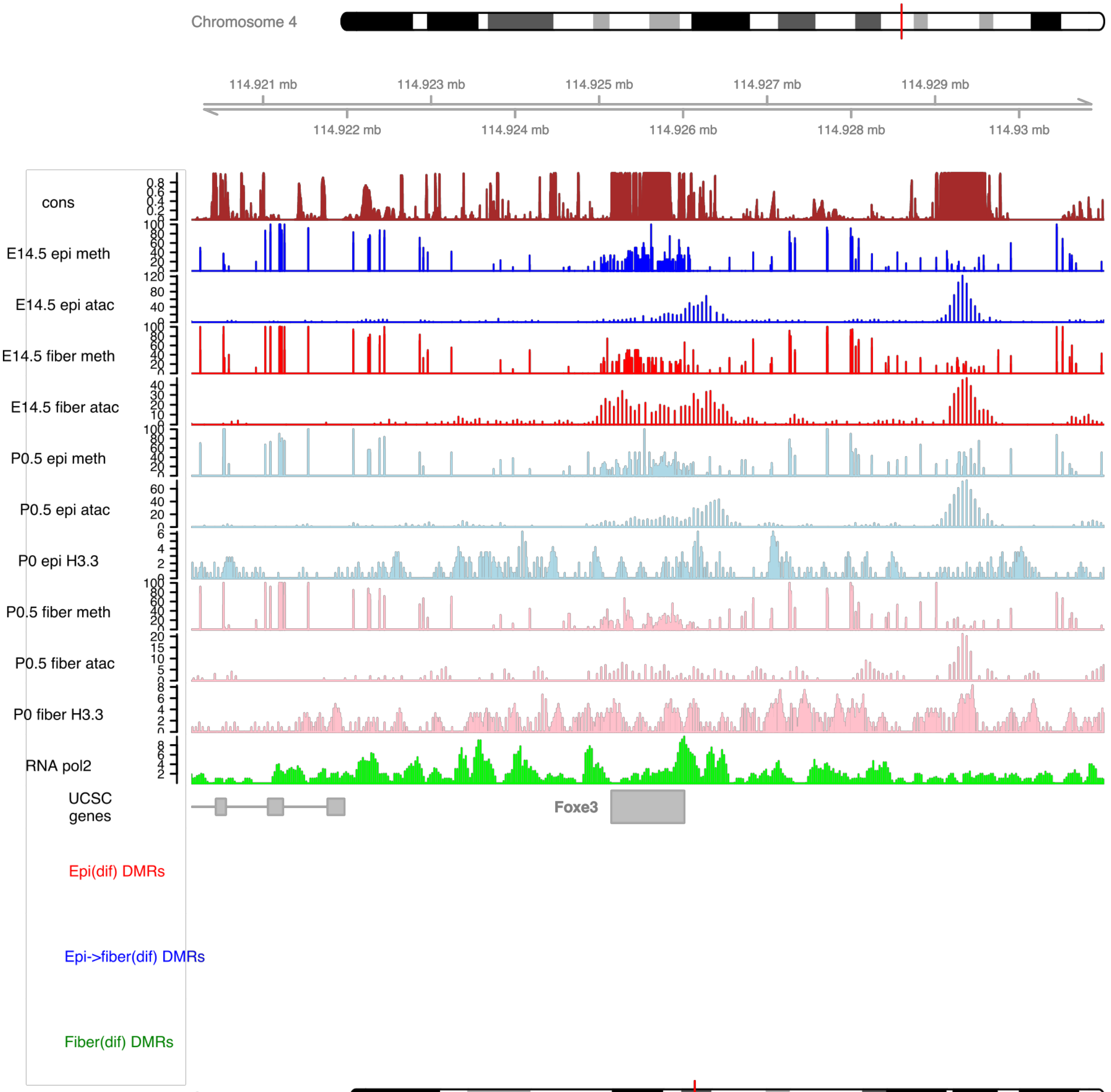
Chromosome 4
114.921 mb
114.923 mb
114.925 mb
114.927 mb
114.929 mb
114.922 mb
114.924 mb
114.926 mb
114.928 mb
114.93 mb
cons
E14.5 epi meth
E14.5 epi atac
E14.5 fiber meth
E14.5 fiber atac
P0.5 epi meth
P0.5 epi atac
P0 epi H3.3
P0.5 fiber meth
P0.5 fiber atac
P0 fiber H3.3
RNA pol2
UCSC genes
Foxe3
Epi(dif) DMRs
Epi->fiber(dif) DMRs
Fiber(dif) DMRs

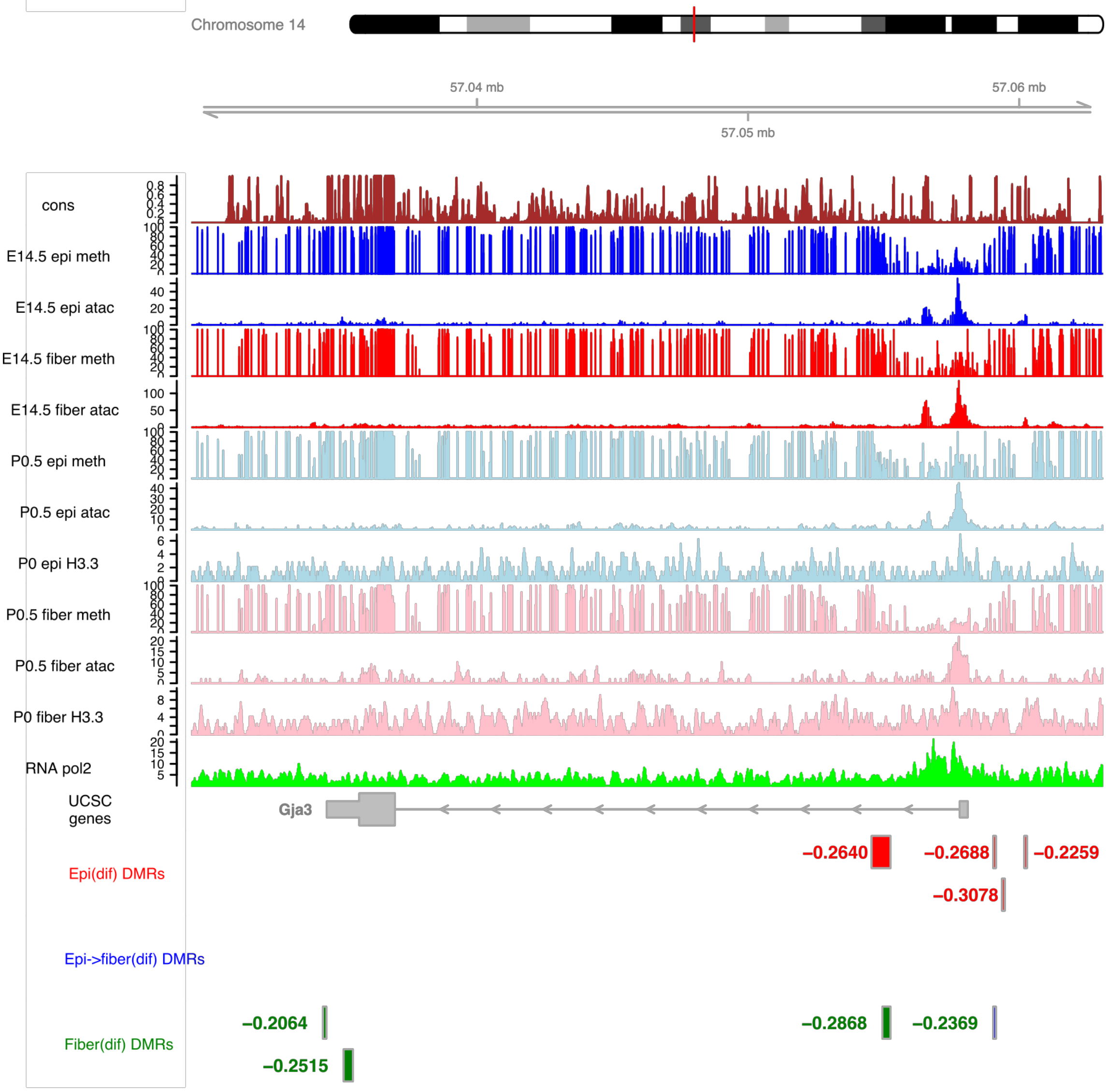
Chromosome 14
57.04 mb
57.06 mb
57.05 mb
cons
E14.5 epi meth
E14.5 epi atac
E14.5 fiber meth
E14.5 fiber atac
P0.5 epi meth
P0.5 epi atac
P0 epi H3.3
P0.5 fiber meth
P0.5 fiber atac
P0 fiber H3.3
RNA pol2
UCSC genes
Gja3
−0.2640
−0.2688
−0.2259
−0.3078
Epi(dif) DMRs
Epi->fiber(dif) DMRs
−0.2064
−0.2868
−0.2369
−0.2515
Fiber(dif) DMRs

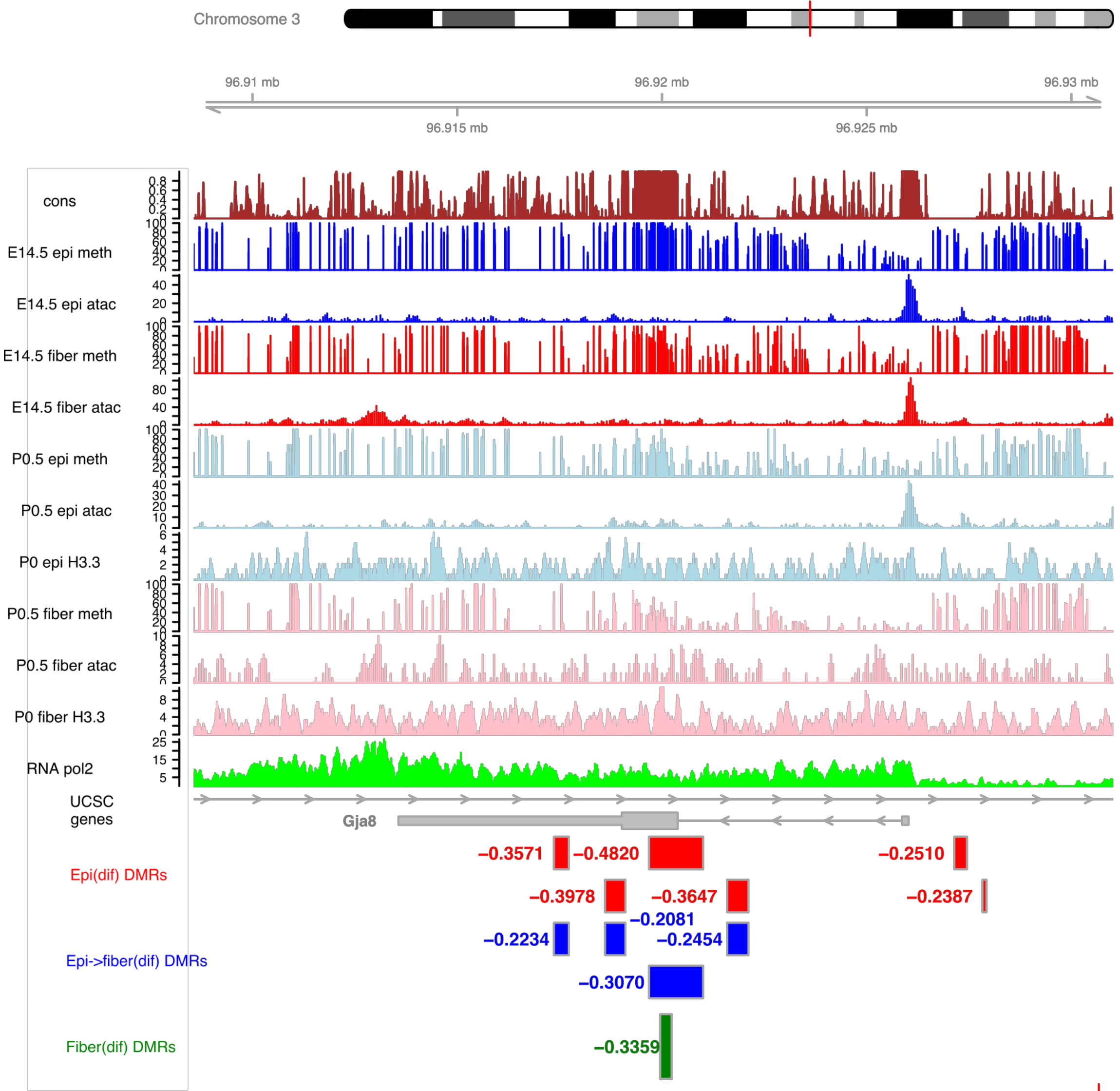
Chromosome 3
96.91 mb
96.92 mb
96.93 mb
96.915 mb
96.925 mb
cons
E14.5 epi meth
E14.5 epi atac
E14.5 fiber meth
E14.5 fiber atac
P0.5 epi meth
P0.5 epi atac
P0 epi H3.3
P0.5 fiber meth
P0.5 fiber atac
P0 fiber H3.3
RNA pol2
UCSC genes
Gja8
Epi(dif) DMRs
−0.3571
−0.4820
−0.2510
−0.3978
−0.3647
−0.2387
Epi->fiber(dif) DMRs
−0.2081
−0.2234
−0.2454
−0.3070
Fiber(dif) DMRs
−0.3359

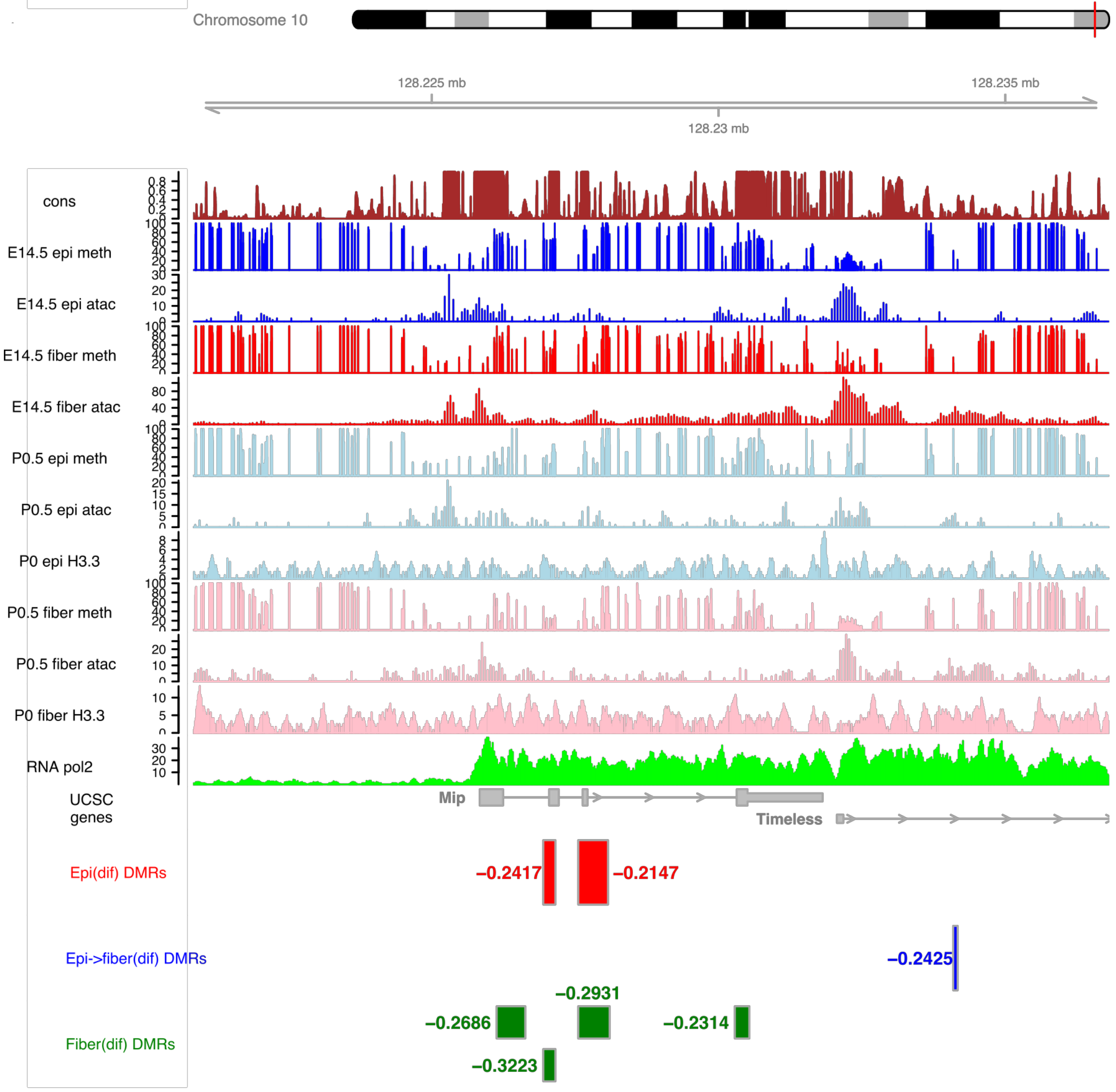
Chromosome 10
128.225 mb
128.235 mb
128.23 mb
cons
E14.5 epi meth
E14.5 epi atac
E14.5 fiber meth
E14.5 fiber atac
P0.5 epi meth
P0.5 epi atac
P0 epi H3.3
P0.5 fiber meth
P0.5 fiber atac
P0 fiber H3.3
RNA pol2
UCSC genes
Mip
Timeless
Epi(dif) DMRs
−0.2417
−0.2147
Epi->fiber(dif) DMRs
−0.2425
Fiber(dif) DMRs
−0.2931
−0.2686
−0.2314
−0.3223
