# Supplementary material for: Dynamic changes in whole genome DNA methylation, chromatin and gene expression during mouse lens differentiation
Source: Epigenetics Chromatin. 2023 Jan 25;16:4. doi: 10.1186/s13072-023-00478-7 (PMC9875507; doi:10.1186/s13072-023-00478-7)
Supplement: Supplementary file 9 — Additional file 9: Table S7. Complete output of HOMER de novo motif search of path Epi(E14.5)Fiber (P0.5)(dif) hypomethylated DMRs. [file 13072_2023_478_MOESM9_ESM.zip › additional_file_9_table_s7/index.html]

../dmrs/path2\_gte20\_hyper\_only.peaks\_homer\_motifs\_noweight// - Homer de novo Motif Results


# Homer *de novo* Motif Results (../dmrs/path2\_gte20\_hyper\_only.peaks\_homer\_motifs\_noweight//)

Known Motif Enrichment Results  
Gene Ontology Enrichment Results  
If Homer is having trouble matching a motif to a known motif, try copy/pasting the matrix file into
STAMP  
More information on motif finding results: HOMER
| Description of Results
| Tips
  
Total target sequences = 3080  
Total background sequences = 38284  
\* - possible false positive  

|  |  |  |  |  |  |  |  |  |
| --- | --- | --- | --- | --- | --- | --- | --- | --- |
| Rank | Motif | P-value | log P-pvalue | % of Targets | % of Background | STD(Bg STD) | Best Match/Details | Motif File |
| 1 |  | 1e-52 | -1.216e+02 | 22.37% | 12.38% | 333.0bp (150.1bp) | NF1(CTF)/LNCAP-NF1-ChIP-Seq(Unpublished)/Homer(0.668) More Information | Similar Motifs Found | motif file (matrix) |
| 2 |  | 1e-40 | -9.221e+01 | 41.53% | 30.17% | 367.0bp (149.0bp) | POL009.1\_DCE\_S\_II/Jaspar(0.787) More Information | Similar Motifs Found | motif file (matrix) |
| 3 |  | 1e-38 | -8.888e+01 | 27.31% | 17.76% | 375.9bp (142.3bp) | MafF(bZIP)/HepG2-MafF-ChIP-Seq(GSE31477)/Homer(0.673) More Information | Similar Motifs Found | motif file (matrix) |
| 4 |  | 1e-38 | -8.844e+01 | 19.64% | 11.49% | 346.7bp (135.9bp) | MA0090.1\_TEAD1/Jaspar(0.835) More Information | Similar Motifs Found | motif file (matrix) |
| 5 |  | 1e-38 | -8.760e+01 | 33.51% | 23.19% | 353.3bp (149.0bp) | MA0512.1\_Rxra/Jaspar(0.590) More Information | Similar Motifs Found | motif file (matrix) |
| 6 |  | 1e-36 | -8.484e+01 | 18.15% | 10.46% | 389.7bp (144.0bp) | MA0161.1\_NFIC/Jaspar(0.819) More Information | Similar Motifs Found | motif file (matrix) |
| 7 |  | 1e-36 | -8.321e+01 | 33.93% | 23.82% | 338.0bp (148.6bp) | MA0259.1\_HIF1A::ARNT/Jaspar(0.822) More Information | Similar Motifs Found | motif file (matrix) |
| 8 |  | 1e-35 | -8.101e+01 | 37.86% | 27.49% | 368.4bp (134.5bp) | Fox:Ebox(Forkhead,bHLH)/Panc1-Foxa2-ChIP-Seq(GSE47459)/Homer(0.680) More Information | Similar Motifs Found | motif file (matrix) |
| 9 |  | 1e-31 | -7.239e+01 | 24.68% | 16.37% | 372.3bp (141.1bp) | PB0098.1\_Zfp410\_1/Jaspar(0.687) More Information | Similar Motifs Found | motif file (matrix) |
| 10 |  | 1e-28 | -6.515e+01 | 23.67% | 15.91% | 370.3bp (134.6bp) | Pitx1(Homeobox)/Chicken-Pitx1-ChIP-Seq(GSE38910)/Homer(0.708) More Information | Similar Motifs Found | motif file (matrix) |
| 11 |  | 1e-24 | -5.670e+01 | 12.89% | 7.51% | 325.4bp (138.4bp) | PB0099.1\_Zfp691\_1/Jaspar(0.752) More Information | Similar Motifs Found | motif file (matrix) |
| 12 |  | 1e-21 | -4.930e+01 | 14.09% | 8.80% | 347.9bp (125.9bp) | MA0496.1\_MAFK/Jaspar(0.666) More Information | Similar Motifs Found | motif file (matrix) |
| 13 |  | 1e-20 | -4.630e+01 | 3.44% | 1.20% | 366.1bp (147.6bp) | MA0154.2\_EBF1/Jaspar(0.614) More Information | Similar Motifs Found | motif file (matrix) |
| 14 |  | 1e-19 | -4.519e+01 | 14.06% | 8.97% | 346.9bp (122.2bp) | PH0138.1\_Pitx2/Jaspar(0.825) More Information | Similar Motifs Found | motif file (matrix) |
| 15 |  | 1e-17 | -3.951e+01 | 2.76% | 0.93% | 375.5bp (144.7bp) | PB0051.1\_Osr2\_1/Jaspar(0.605) More Information | Similar Motifs Found | motif file (matrix) |
| 16 |  | 1e-16 | -3.902e+01 | 3.41% | 1.32% | 383.2bp (148.4bp) | PB0046.1\_Mybl1\_1/Jaspar(0.712) More Information | Similar Motifs Found | motif file (matrix) |
| 17 |  | 1e-16 | -3.887e+01 | 1.04% | 0.14% | 260.2bp (139.6bp) | MA0119.1\_TLX1::NFIC/Jaspar(0.762) More Information | Similar Motifs Found | motif file (matrix) |
| 18 |  | 1e-15 | -3.577e+01 | 2.95% | 1.11% | 282.5bp (127.3bp) | PB0051.1\_Osr2\_1/Jaspar(0.646) More Information | Similar Motifs Found | motif file (matrix) |
| 19 |  | 1e-12 | -2.890e+01 | 0.42% | 0.02% | 298.9bp (98.2bp) | MA0155.1\_INSM1/Jaspar(0.617) More Information | Similar Motifs Found | motif file (matrix) |
| 20 |  | 1e-12 | -2.790e+01 | 9.12% | 5.89% | 319.0bp (128.0bp) | MA0030.1\_FOXF2/Jaspar(0.722) More Information | Similar Motifs Found | motif file (matrix) |
| 21 \* |  | 1e-11 | -2.741e+01 | 0.39% | 0.02% | 225.5bp (97.6bp) | Nr5a2(NR)/mES-Nr5a2-ChIP-Seq(GSE19019)/Homer(0.627) More Information | Similar Motifs Found | motif file (matrix) |
| 22 \* |  | 1e-11 | -2.680e+01 | 1.43% | 0.40% | 283.9bp (125.0bp) | PB0168.1\_Sox14\_2/Jaspar(0.671) More Information | Similar Motifs Found | motif file (matrix) |
| 23 \* |  | 1e-9 | -2.227e+01 | 1.33% | 0.41% | 229.7bp (105.1bp) | PB0178.1\_Sox8\_2/Jaspar(0.655) More Information | Similar Motifs Found | motif file (matrix) |
| 24 \* |  | 1e-9 | -2.220e+01 | 0.26% | 0.01% | 94.6bp (41.2bp) | MA0009.1\_T/Jaspar(0.632) More Information | Similar Motifs Found | motif file (matrix) |
| 25 \* |  | 1e-9 | -2.173e+01 | 0.36% | 0.03% | 198.4bp (101.7bp) | ZFX(Zf)/mES-Zfx-ChIP-Seq(GSE11431)/Homer(0.653) More Information | Similar Motifs Found | motif file (matrix) |
| 26 \* |  | 1e-8 | -1.869e+01 | 0.23% | 0.01% | 273.3bp (47.4bp) | PB0046.1\_Mybl1\_1/Jaspar(0.690) More Information | Similar Motifs Found | motif file (matrix) |
| 27 \* |  | 1e-7 | -1.687e+01 | 0.26% | 0.02% | 179.3bp (118.9bp) | POL008.1\_DCE\_S\_I/Jaspar(0.650) More Information | Similar Motifs Found | motif file (matrix) |
| 28 \* |  | 1e-5 | -1.210e+01 | 0.16% | 0.01% | 112.8bp (72.1bp) | PB0130.1\_Gm397\_2/Jaspar(0.566) More Information | Similar Motifs Found | motif file (matrix) |
| 29 \* |  | 1e-4 | -9.596e+00 | 0.29% | 0.05% | 195.9bp (79.2bp) | PB0173.1\_Sox21\_2/Jaspar(0.720) More Information | Similar Motifs Found | motif file (matrix) |
