# Supplementary material for: Dynamic changes in whole genome DNA methylation, chromatin and gene expression during mouse lens differentiation
Source: Epigenetics Chromatin. 2023 Jan 25;16:4. doi: 10.1186/s13072-023-00478-7 (PMC9875507; doi:10.1186/s13072-023-00478-7)
Supplement: Supplementary file 9 — Additional file 9: Table S7. Complete output of HOMER de novo motif search of path Epi(E14.5)Fiber (P0.5)(dif) hypomethylated DMRs. [file 13072_2023_478_MOESM9_ESM.zip › additional_file_9_table_s7/homerResults/motif20.similar.html]

motif20

## Information for motif20

  
Reverse Opposite:
